# A review of the indigenous coffee resources of Uganda and their potential for coffee sector sustainability and development

**DOI:** 10.3389/fpls.2022.1057317

**Published:** 2023-02-17

**Authors:** Aaron P. Davis, Catherine Kiwuka, Aisyah Faruk, John Mulumba, James Kalema

**Affiliations:** ^1^ Crops & Global Change, Royal Botanic Gardens, Kew, Richmond, Surrey, United Kingdom; ^2^ Plant Genetic Resources Centre, National Agricultural Research Organization, Entebbe, Uganda; ^3^ Partnerships (Conservation), Millennium Seed Bank (Royal Botanic Gardens, Kew), Wakehurst, Sussex, United Kingdom; ^4^ Department of Plant Sciences, Microbiology and Biotechnology, College of Natural Sciences, Makerere University, Kampala, Uganda

**Keywords:** genetic resources, climate change, Uganda, excelsa coffee, conservation, robusta coffee, coffee, Crop Wild Relative (CWR)

## Abstract

Uganda is a major global coffee exporter and home to key indigenous (wild) coffee resources. A comprehensive survey of Uganda’s wild coffee species was undertaken more than 80 years ago (in 1938) and thus a contemporary evaluation is required, which is provided here. We enumerate four indigenous coffee species for Uganda: *Coffea canephora*, *C. eugenioides*, *C. liberica* (var. *dewevrei*) and *C. neoleroyi*. Based on ground point data from various sources, survey of natural forests, and literature reviews we summarise taxonomy, geographical distribution, ecology, conservation, and basic climate characteristics, for each species. Using literature review and farm survey we also provide information on the prior and exiting uses of Uganda’s wild coffee resources for coffee production. Three of the indigenous species (excluding *C. neoleroyi*) represent useful genetic resources for coffee crop development (e.g. *via* breeding, or selection), including: adaptation to a changing climate, pest and disease resistance, improved agronomic performance, and market differentiation. Indigenous *C. canephora* has already been pivotal in the establishment and sustainability of the robusta coffee sector in Uganda and worldwide, and has further potential for the development of this crop species. *Coffea liberica* var. *dewevrei* (excelsa coffee) is emerging as a commercially viable coffee crop plant in its own right, and may offer substantial potential for lowland coffee farmers, i.e. in robusta coffee growing areas. It may also provide useful stock material for the grafting of robusta and Arabica coffee, and possibly other species. Preliminary conservation assessments indicate that *C. liberica* var. *dewevrei* and *C. neoleroyi* are at risk of extinction at the country-level (Uganda). Adequate protection of Uganda’s humid forests, and thus its coffee natural capital, is identified as a conservation priority for Uganda and the coffee sector in general.

## Introduction

Uganda is the world’s seventh largest exporter of coffee, and Africa’s second largest exporter, after Ethiopia. In 2019/20 Uganda exported c. 330,540 metric tons (International Coffee Organization (ICO), 2022) of robusta (*Coffea canephora*) and Arabica (*C. arabica*) coffee, at an estimated ratio of around 4:1, respectively (Uganda Coffee Development Authority (UCDA), 2017). Uganda is now the fourth largest robusta producer in the world, after Vietnam, Brazil and Indonesia (Uganda Coffee Development Authority (UCDA) 2017). Coffee accounts for c. 15% of Uganda’s annual export revenue, with c. 4.2% of the population (1.7 m people) engaged in coffee farming (Uganda Coffee Development Authority (UCDA), 2017), and c. 20% (8 m people) working in the coffee sector (Kiwuka et al., 2021). Despite its success, the Ugandan coffee sector faces major challenges, which are set to accelerate over the coming decades, due to climate change and other disruptive influences. Uganda is fortunate, however, in possessing key wild (indigenous) coffee genetic resources, which offer promise for coffee crop development, climate-resilience potential (Kiwuka et al., 2021) and commercial enrichment.

Wild coffee (genetic) resources, both from within the two major crop species, *C. arabica* and *C. canephora*, and other species, have played a vital role in sustaining coffee production (farming) and thus the sector as whole (Davis et al., 2019). Examples include the use of wild material for: coffee berry disease (CBD; *Colletotrichum kahawae* J.M.Walter & Bridge) resistance for Ethiopian *C. arabica* (Yonas et al., 2014); coffee wilt disease (CWD; *Gibberella xylarioides* R. Heim & Sacca) resistance for Ugandan *C. canephora* (Kiwuka et al., 2021; Mulindwa et al., 2022); coffee leaf rust (CLR; *Hemileia vastatrix* Berk. & Broome) resistance, globally, for *C. arabica*, through crossing with *C. canephora* (Clarindo et al., 2013; Avelino et al., 2015) and *C. liberica* (Narasimhaswamy, 1960; Surya Prakash et al., 2002); and coffee leaf miner (*Perileucoptera coffeella* Méneville) resistance (Medina Filho et al., 1977a; Medina Filho et al., 1977b) and drought tolerance (Grisi et al., 2008; Melo et al., 2014; Carvalho et al., 2017) in *C. arabica*, through crossing with *C. racemosa* (Davis et al., 2021a). It is worth noting that wild species were used to sustain the global coffee industry in response to the devasting influence of CLR at the end of nineteenth century, firstly using *C. liberica*, from c. 1875–1900, and then *C. canephora* from the early 1900s onwards (McCook, 2014; Davis et al., 2019; McCook, 2019; Davis et al., 2022). Other coffee species are exported on a small-scale, including: *C. congensis* (Congo coffee) and particularly the hybrid ‘congusta’ (Bharatha Nandhini et al., 2013), *C. eugenioides* (see main text), *C. racemosa* and *C. zanguebariae* (Ibo or Zanzibar coffee) (Davis et al., 2021a). *Coffea stenophylla* was once exported from Upper West Africa (Davis et al., 2020), and may have potential as a crop plant on the basis of being able to provide an Arabica-like flavour at much higher temperatures compared to *C. arabica* (Davis et al., 2021b). Many species are used locally, across Africa, the Indian Ocean Islands (Madagascar and the Mascarene Islands) and Asia, as a substitute for *C. arabica* (Cheney, 1925; Davis et al., 2006). There could be further potential for coffee crop plant development (Davis et al., 2019) amongst the 130 species of *Coffea* now known to science (Davis and Rakotonasolo, 2021).

The most recent (and only) review of Ugandan wild coffee genetic resources was undertaken by A.S. Thomas in 1938, and published six years afterwards (Thomas, 1944). Thomas (1944) included four species in his review *C. eugenioides*, *C. canephora*, *C. excelsa* (now known as *C. liberica* var. *dewevrei*), and *C. spathicalyx*, although the last of these species has since been transferred to the genus *Calycosiphonia* (*C. spathicalyx*) (Davis et al., 2006). Despite the abundance of useful information in the review of Thomas (1944), much has changed since 1938 and more data is now available. In this contribution, we undertake a contemporary review of indigenous coffee resources, with an emphasis on their potential for coffee sector sustainability. We use ground point data from various sources (herbarium specimens and occurrence databases), survey of natural forests (2015 to 2022), and literature reviews, to summarise the taxonomy (and common names), geographical distribution, ecology, conservation, and basic climate characteristics, for each species. Using literature review and farm survey (2015 to 2022), we also provide information on the prior, existing, and potential future uses of Uganda’s wild coffee resources for coffee production.

Our objectives were to review: (1) the eco-geography and climate requirements of Uganda’s indigenous coffee species; (2) the extinction risk of these species at the country level; and (3) the application of this natural capital for the development and sustainability of the coffee sector.

## Methods

### Use of scientific names

Scientific names follow the accepted nomenclature for *Coffea*, based on peer-reviewed taxonomic and systematic research, as summarised in global plant name checklists (Govaerts et al., 2022). Synonyms for species names and other taxa are not included here, but are available from other sources (Davis et al., 2006; Govaerts et al., 2022).

### Data for mapping, climate profiling and conservation assessments

Occurrence data points derived from herbarium specimens and field surveys (see below) were used to provide the data for the production of distribution maps and basic climate profiling analyses for *C. canephora*, *C. eugenioides*, *C. liberica* var. *dewevrei* and *C. neoleroyi*. We consulted herbarium specimen records from seven herbaria (BM, BR, K, MHU, P, WAG); herbarium codes follow (Holmgren et al., 1990; Thiers, 2019). Location data were georeferenced (if lacking coordinates), manually checked for geolocation accuracy (1 km or less) using GoogleEarth^®^ and corrected if necessary. The herbarium and field surveys provided a dataset of 583 records, comprising, 275 for *C. canephora*, 198 for *C. eugenioides*, 109 for *C. liberica* var. *dewevrei* and 1 for *C. neoleroyi*.

### Fieldwork and other data

Study of wild populations of *C. canephora*, *C. liberica* var. *dewevrei*, and *C. eugenioides*, and farm study visits for *C. canephora* and *C. liberica* var. *dewevrei*, were undertaken between 2015 and 2022. Location, habitat and ecology data were collected from forest sites, and basic agronomy observations were made during the farm visits. Herbarium specimens (see above) and literature were consulted for additional information (including habitat, vegetation, uses and vernacular names). Information on the global distribution of coffee species was taken from published sources (Davis et al., 2006; Davis et al., 2019).

### Mapping

**Figure 1:**
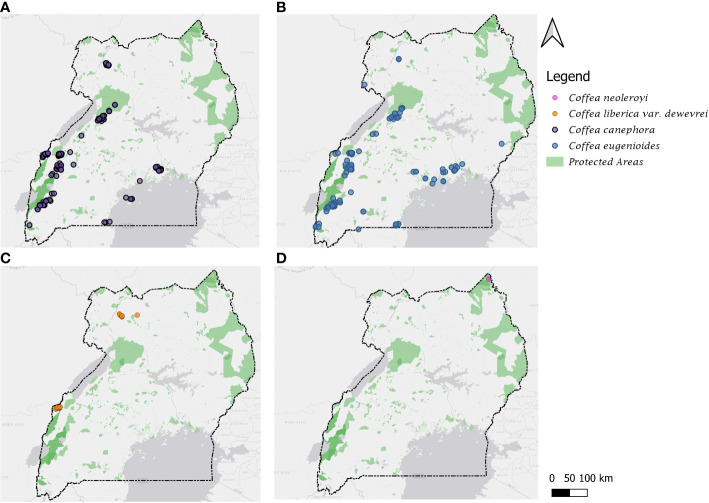
Distribution maps for the four indigenous *Coffea* species of Uganda. **(A)** *C. canephora*; **(B)** *C. eugenioides*; **(C)** *C. liberica* var. *dewevrei*; **(D)** *C. neoleroyi*. Protected areas based on World Database on Protected Areas (see Methods).

A total of 583 data records were used to produce the distribution maps (**Figure 1**) for the four species (see Data for Mapping, Climate Profiling and Conservation Assessments). The maps were produced in QGIS 3.16 (QGIS Development Team, 2022), using the ESRI Gray (light) basemap available through the QuickMapServices 19.11.1 version plugin (NextGIS, 2019) and administrative area boundaries from GDAM version 1.0 (https://gadm.org/). For mapping the protected areas, we used the World Database on Protected Areas data, obtained *via* the Protected Planet portal (https://www.protectedplanet.net/en/thematic-areas/wdpa?tab=WDPA) [accessed November 2022].

### Conservation assessments

To produce preliminary national conservation assessments, we followed the IUCN Red List of Threatened Species criteria (IUCN Standards and Petitions Subcommittee, 2022). Area-based conservation metrics were generated from the mapping data set of 583 records (see above) using GeoCat (Bachman et al., 2011) GeoCAT (kew.org) [accessed 1 September 2022] with default settings (Area of Occurrence (AOO) cell width of 2 km).

### Climate profiling analyses

**Figure 2:**
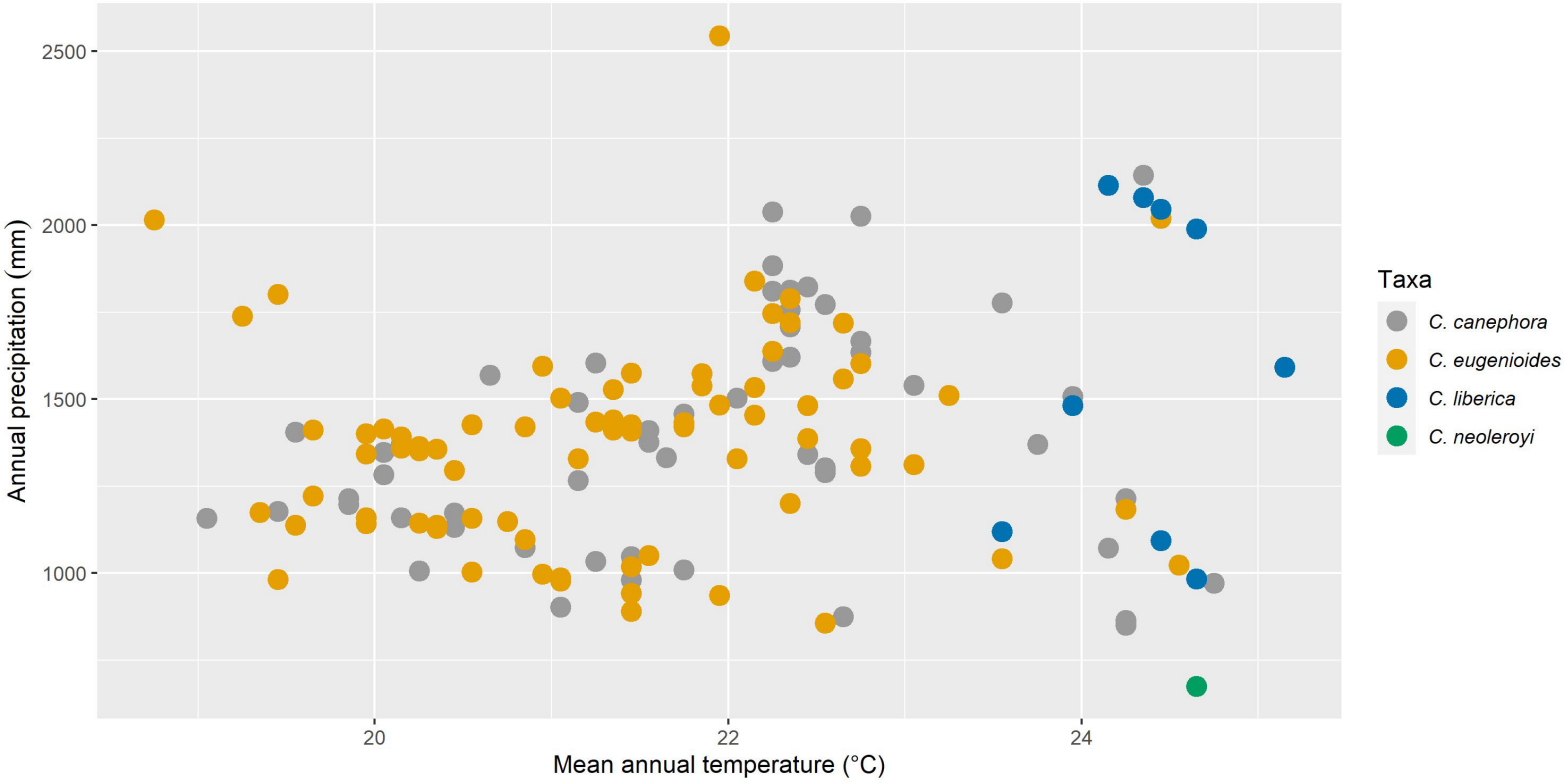
Scatter plots of modelled annual mean temperature (CHELSA Bio1) vs. total mean annual precipitation (CHELSA Bio12) for four indigenous Ugandan *Coffea* species (mean values in parentheses): *C. canephora* (21.8°C/1,389 mm); *C. eugenioides* (21.3°C/1,370 mm); *C. liberica* var. *dewevrei* (24.4°C/1,560 mm); and *C. neoleroyi* (data not available).

**Figure 3:**
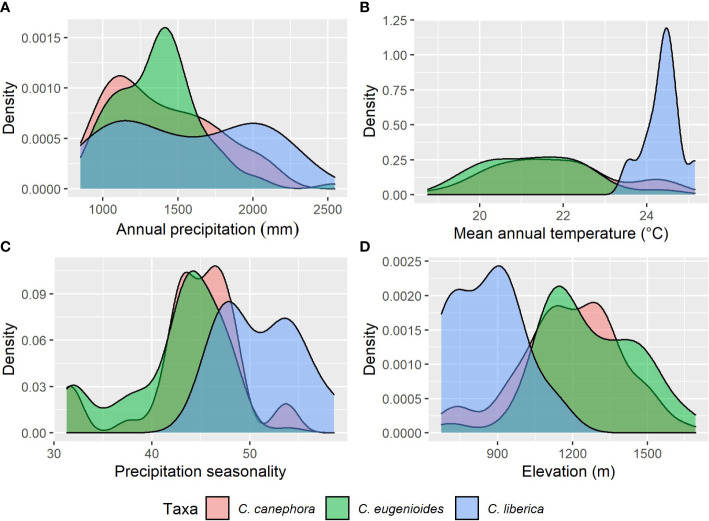
Density plots based for key climate variables and elevation (see Methods) for *C. canephora*, *C. eugenioides*, and *C. liberica* var. *dewevrei*. **(A)** total mean annual precipitation (mm year) from CHELSA Bio1; **(B)** mean annual temperature (°C) from CHELSA Bio12; **(C)** precipitation seasonality from CHELSA Bio15; **(D)** elevation (m). Summary statistics: (mean values in parentheses): *C. canephora* (21.8°C/1,389 mm/44/1187 m); *C. eugenioides* (21.3°C/1,370 mm/42/1261 m); and *C. liberica* var. *dewevrei* (24.4°C/1,560 mm/51/857 m). See **Table 1**.

We resampled all specimen data to remove duplicates within 1 km of each other, reducing the total number of records used from 583 to 176 (85 for *C. canephora*, 80 for *C. eugenioides*, 10 for *C. liberica* var. *dewevrei* and 1 for *C. neoleroyi*. To understand the fundamental climatic requirements, the statistics package R (R Core Team, 2020) was used to sample specimen data against 19 Bioclim variables (Busby, 1991) from the CHELSA dataset (Karger et al., 2017). For our overview of climatic parameters, we selected the following three Bioclims: Bio1 Annual mean temperature, Bio12 Annual Precipitation, and Bio15 Precipitation Seasonality. Of the 19 Bioclim variables (Busby, 1991), these three have been shown to provide a pragmatic summary of basic climate requirements for *Coffea* (Davis et al., 2021a; Davis et al., 2021b); and are included amongst the key drivers of modelled coffee distribution (Moat et al., 2017; Moat et al., 2019). Scatter (**Figure 2**) and density (**Figure 3**) plots were plotted using R (R Core Team, 2020), using the ggplot2 (Wickham, 2016) and ggpubr packages (Kassambara, 2020). These modelling methods have been shown to provide climate metrics that are similar to those provided for coffee species in cultivation (including farmed conditions) and in the wild, produced by direct measurement and other means (Davis et al., 2021b). For validation purposes, our modelled mean annual temperatures (from Bio1), total annual precipitation (Bio12) and precipitation seasonality (Bio15), were compared against publicly available monthly mean temperature precipitation charts for Uganda and published data for cultivated *C. canephora* (DaMatta and Ramalho, 2006; Kath et al., 2020; Venancio et al., 2020); published data are not available for the three other species studied here.

## Results

### Survey of wild (indigenous) Ugandan coffee species

We enumerate four indigenous (wild) coffee species for Uganda: *C. canephora*, *C. eugenioides*, *C. liberica* var. *dewevrei* and *C. neoleroyi*. For each species, this survey includes: the global distribution and global IUCN conservation assessment; and for Uganda, the distribution, ecology, elevation, preliminary conservation assessment, protected area occurrence, common names, uses (other than beverage) and miscellaneous notes.

### Key to Ugandan coffee (*Coffea*) species

A. Leaves deciduous and/or restricted to short branches; flowers appearing terminal on short shoots; corolla tube much longer than wide …………………………………….. 4. *C. neoleroyi*
A. Leaves evergreen, distributed evenly along the side branches; flowers distinctly axillary; corolla tube shorter or not much longer than wide …………………………………………… B
B. Leaves 12–40 × 4.5–22 cm, with 8–17 pairs of secondary veins C
B. Leaves 3–12 × 1–7 cm, with 5–7 pairs of secondary veins 2. *C. eugenioides*
C. Leaves thick (rather leathery), domatia present and usually obvious, on the secondary vein (at the base) or in the secondary vein-midrib axil ……… 3. *C. liberica* var. *dewevrei*
C. Leaves thin (paper-like to slightly leathery), domatia absent or inconspicuous, if present in the secondary vein-midrib axil ………………………………………….. 1. *C. canephora*

**Figure 4:**
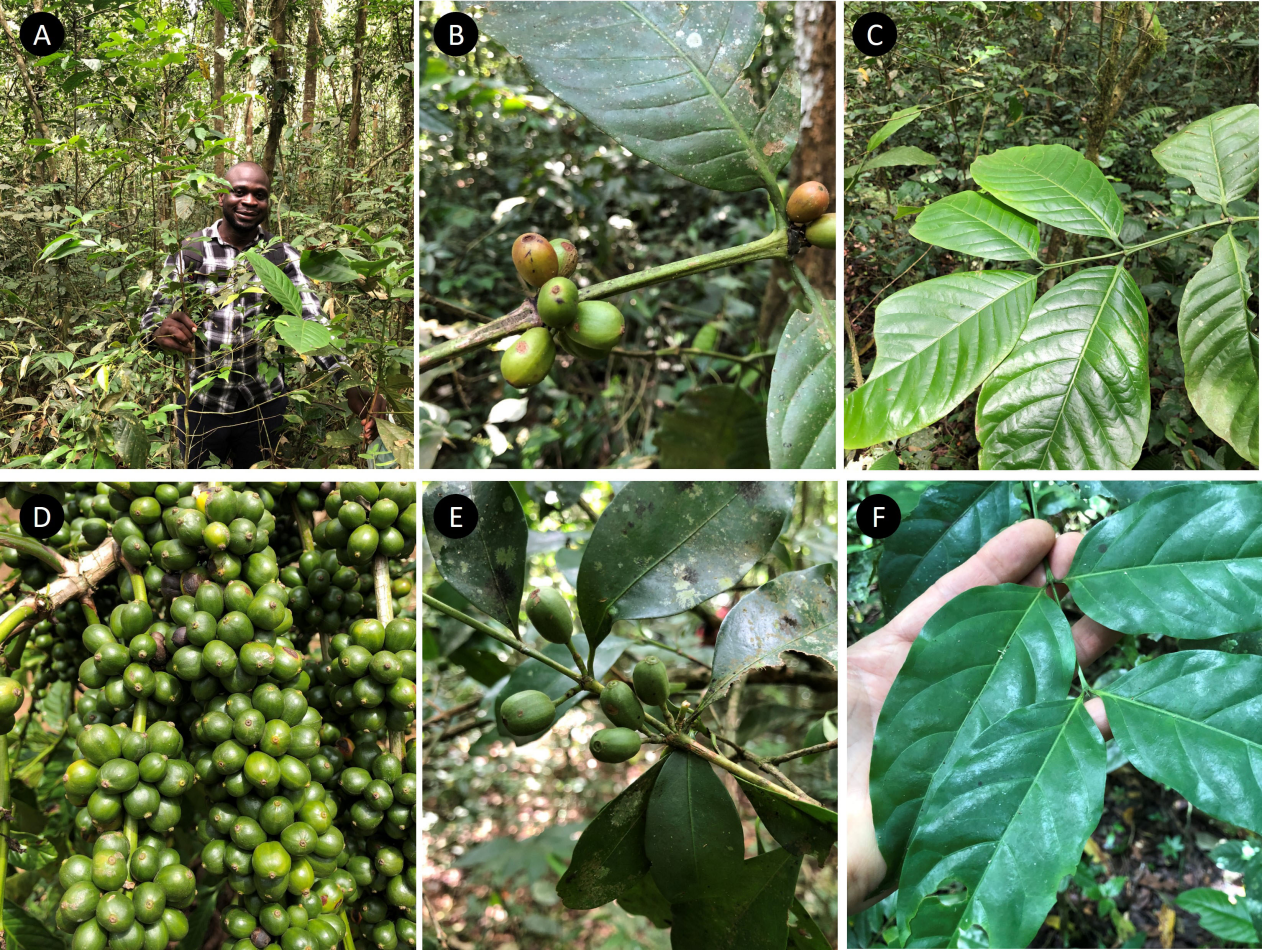
*Coffea canephora* (robusta coffee) and *C. eugenioides*. **(A)** Dr Robert Acidri: right hand holding *C. canephora* and left hand holding *C. eugenioides*; **(B)** *C. canephora*, fruiting shoot (immature); **(C)** *C. canephora*, leaves; **(D)** *C. canephora*, unripe fruiting branch (immature); **(E)** *C. eugenioides*, fruiting branch (immature); **(F)** *C. eugenioides*, larger leaved variant. **(A–C)**, **(E, F)**: Itwara Forest (wild), western Uganda; **(D)** farmed in Masaka, central Uganda.

**Figure 5:**
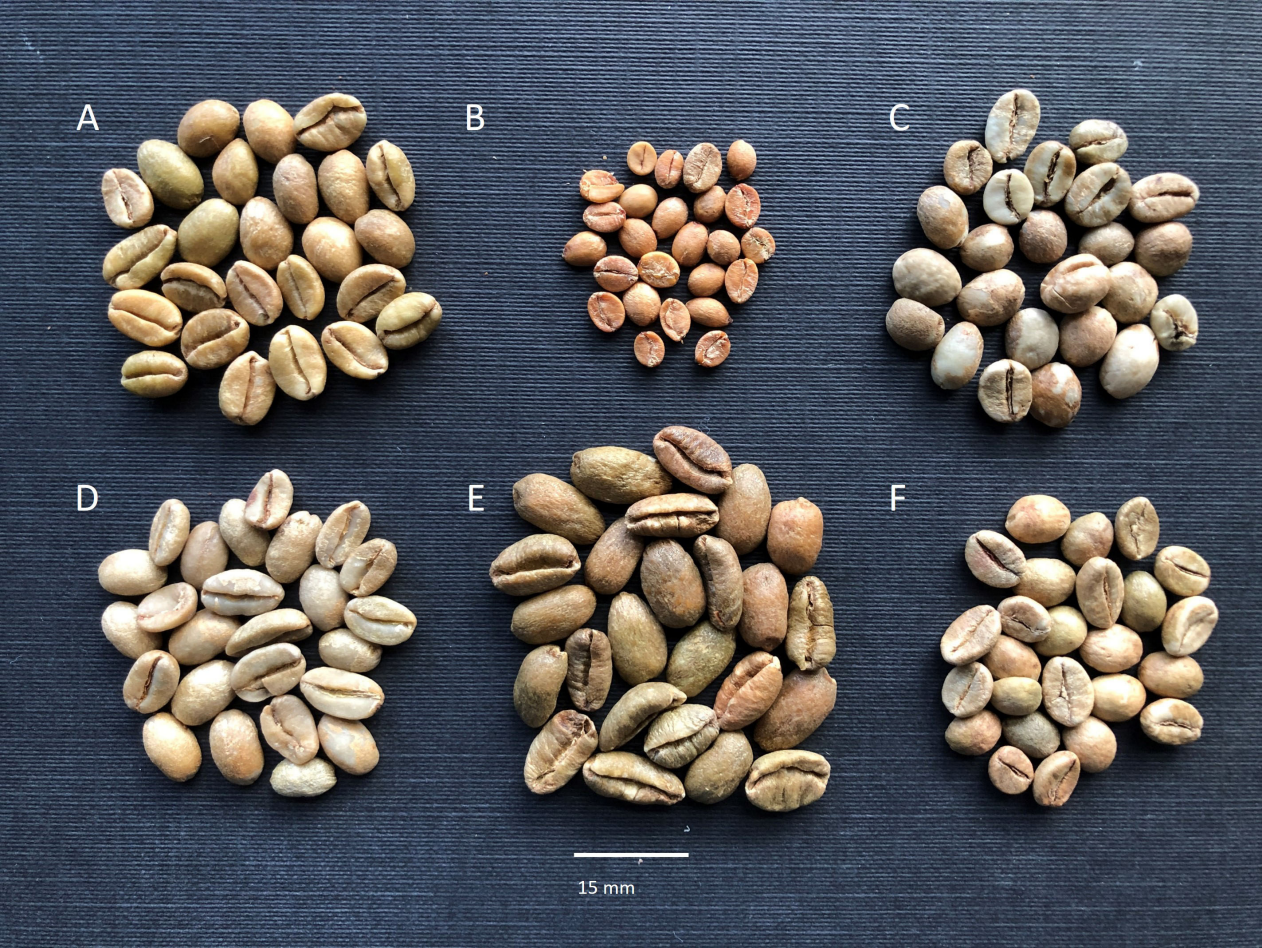
Seeds (unroasted coffee beans) of three Ugandan coffee species, with some cultivated species for size comparison. **(A)** *C. liberica* var. *dewevrei* (excelsa coffee), cultivated in central Uganda; **(B)** *C. eugenioides*, cultivated in Kampala, Uganda (1921), from RBG Kew Economic Botany Collection; **(C)** *C. canephora* (robusta coffee), cultivated in Uganda; **(D)** *C. arabica* (Arabica coffee), cultivated in Ethiopia; **(E)** *C. liberica* var. *liberica* (Liberica or Liberian coffee), cultivated in Malaysia; **(F)** *C. canephora* (robusta coffee), cultivated in India. Each sample comprises 25 seeds.

**1. *Coffea canephora*** Pierre ex A.Froehner, Notizbl. Bot. Gart. Berlin-Dahlem 1: 237 (1897) **Figures 4A–D**, **5C**.

*Global distribution:* west Tropical Africa (western Ghana, Guinea, Ivory Coast, Liberia [inferred or observed; no herbarium specimen data known], Nigeria); west-central Tropical Africa (Cabinda, Cameroon, Congo, Central African Republic, Democratic Republic of Congo, Gabon); north-east Angola. Tropical Africa (southern South Sudan); east Tropical Africa (Tanzania, Uganda); south Tropical Africa (northern Angola). The exact limit of natural distribution is difficult to ascertain owing to introduction and naturalisation. Widely cultivated as robusta coffee across the tropical belt of the world and frequently as Conilon in Brazil; naturalised in Tropical Africa and other tropical areas (not listed here).

*Global IUCN conservation assessment:* Least Concern (LC) (Chadburn and Davis, 2017a).

***Information for Uganda***

*Distribution:* Throughout Uganda but mainly in eastern and western parts of the country. Uganda is a centre of diversity for *C. canephora* (Gomez et al., 2009; Cubry et al., 2013; Merot-L'anthoene et al., 2019; Kiwuka et al., 2021), and is the only country on the eastern side of the Great Rift Valley that holds substantial wild populations of this species. **Figure 1A**.

*Ecology:* An exclusively forest-dwelling species, found in the understorey of humid, evergreen forest (rainforest), occurring with a wide range of dominant tree species. Often occurring in the same forests and in close proximity to *C. eugenioides* (**Figures 1**, **4A**) and at low elevations with *C. liberica* var. *dewevrei* (**Figure 1**).

*Elevation:* 655–1570 m (observed and recorded)*;* 675–1660 m (modelled).

*Preliminary country-level IUCN conservation assessment:* Least Concern (LC). The Extent of Occurrence (EOO) is 128,922 km^2^ (LC); and Area of Occurrence (AOO) is 392 km^2^ (Endangered (EN)). Whilst the EOO for *C. canephora* in Uganda is substantial, the AOO calculation falls within the limits for EN, indicating that the preservation of this species should not be taken for granted and that careful monitoring is required. Over most the eastern part of its distribution in Uganda (**Figure 1A**), *C. canephora* is now restricted to smaller and increasingly fragmented forests; if these populations were extirpated, the EOO would be severely reduced (c. 33,102 km^2^) and the EOO-based rating increased to Near Threatened (NT). Populations in the larger protected areas appear to be healthy, with a high density of individuals, but encroachment, deforestation and disturbance in some protected areas (e.g. Zoka and Itwara Central Forest Reserves) are negatively affecting AOO, number of mature individuals, habitat quality, and population health.

*Main protected area occurrence:* Budongo (CFR), Bugoma (CFR), Itwara (CFR), Kagombe (CFR), Kalinzu (CFR), Kasyoha - Kitomi (CFR), Kibale (NP), Kisangi (CFR), Mabira (CFR), Murchison Falls (NP), Queen Elizabeth National Park (NP/BR), Rwensama (CFR), Semuliki (NP), South Maramagambo (CFR), Tero (CFR), Zoka (CFR). Key: Central Forest Reserve (CFR), National Park (NP), UNESCO-MAB Biosphere Reserve (BR).

*Ugandan names:* From (Eggeling and Dale, 1952): Mwanyi (Luganda, Lutoro, Kuamba, Lunyoro). From (Kalema and Hamilton, 2020): Mwanyi (Kwamba, Rutooro), Mumwanyi (Luganda), Omwanyi (Runyoro).

*Other names:* Wild robusta Coffee (Eggeling and Dale, 1952; Kalema and Hamilton, 2020).

*Uses (other than beverage):* As a masticatory-stimulant (due to the presence of caffeine), and snack, either fresh or dried. A traditional usage is to take a small number (c. 10) of prepared fruits (unripe or semi-ripe and dried whole, sometimes boiled in water) and package them in dried banana leaves, for sale in local shops and at roadsides (personal observation and Thomas (1944). *Coffea canephora* is used in various traditional and ritualistic activities, as an emblem for brotherhood and deep friendship. Even though this activity is steadily declining, it is still used in traditional marriage ceremonies in the Buganda culture.

**2. *Coffea eugenioides*** S.Moore, J. Bot. 45: 43 (1907) **Figures 4E, F**, **5C**.

*Global distribution:* west-central Tropical Africa (Burundi [inferred or observed; no herbarium specimen data known], Rwanda, eastern Democratic Republic of Congo); north-east Tropical Africa (southern South Sudan); east Tropical Africa (central & eastern Kenya, eastern Tanzania, Uganda).

*Global IUCN conservation assessment:* Least Concern (LC) (O'Sullivan et al., 2020).

***Information for Uganda***

*Distribution:* Throughout Uganda but mainly in eastern and western parts of the country. Uganda is a centre of diversity for *C. eugenioides* (Thomas, 1944) and is the only country on the eastern side of the Great Rift Valley that holds substantial wild populations of this species. **Figure 1B**.

*Ecology:* An exclusively forest-dwelling species, found in the understorey of humid, evergreen forest (rainforest), occurring with a wide range of dominant tree species. Often occurring in the same forests and in close proximity to *C. canephora* (**Figures 1**, **4A**) and at low elevations with *C. liberica* var. *dewevrei* (**Figure 1**).

*Elevation:* 910*–*1828 m (observed and recorded); 700*–*1693 m (modelled).

*Preliminary country-level IUCN conservation assessment:* Least Concern (LC). The Extent of Occurrence (EOO) is 133,516 km^2^ (LC); and Area of Occurrence (AOO) is 372 km^2^ (Endangered (EN). Whilst the EOO for *C. eugenioides* in Uganda is substantial, the AOO calculation returns a rating of EN, indicating that the preservation of this species should not be taken for granted and that careful monitoring is required. Over most the eastern part of its distribution in Uganda (**Figure 1B**), *C. eugenioides* is now restricted to smaller and increasingly fragmented forests; if these populations were extirpated the EOO would be severely reduced (c. 39,117 km^2^) and the EOO based rating increased to Near Threatened (NT). Populations in the larger protected areas appear healthy, with a high density of individuals, but encroachment, deforestation, and disturbance in some protected areas (e.g. Zoka Forest and Itwara Central Forest Reserves) are negatively affecting AOO, number of mature individuals, habitat quality and population health.

*Main protected area occurrence:* Budongo (CFR), Bugoma (CFR), Bwindi Impenetrable National Park (NP/WH), Itwara (CFR), Kagombe (CFR), Kalinzu (CFR), Kasyoha - Kitomi (CFR), Kibale (NP), Kitubulu (CFR), Luvunya (CFR), Mabira (CFR), Malabigambo (CFR), Mbale (CFR), Mpanga (CFR), Mukambwe (CFR), Namalala (CFR), North Maramagambo (CFR), Nsube (CFR), Queen Elizabeth National Park (BR/NP), Rwensama (CFR), Semuliki (NP), South Maramagambo (CFR), Zoka (CFR). Key: Central Forest Reserve (CFR), National Park (NP), UNESCO-MAB Biosphere Reserve (BR), World Heritage Site (WH).

*Ugandan names:* From (Katende et al., 1999): Emwanji (Ateso), Mwanji (Adhola, Luganda, Lugwe), Imwanji (Lugisu), Omwani (Rukiga, Runyankore, Runyoro, Rutooro), Nkiga [sic] (Rutooro). From (Kalema and Hamilton, 2020): Mumwanyi (Luganda), Mwanyi (Ganato), Nkinga (Rutooro), Omwanyi (Runyoro).

*Other names:* Mukono coffee (English; (Bullock, 1930) but not widely applied). The name ‘Nandi coffee’ is used in Kenya for *C. eugenioides*, but refers to a Kenyan place name and hence a Kenyan variant of this species.

*Uses (other than beverage):* From (Katende et al., 1999): the (‘sweet and tasty’) ripe fruits are eaten as a snack, mainly by children; eaten in moderate amounts (Batooro, Bamba, Banyankore, Baganda); the fruits may also be boiled, dried and stored for later use as dry snacks (Baganda); dried leaves are put on hot charcoal and the smoke inhaled to relieve headache; the materials for the above uses are collected from the wild and are not cultivated.

**3. *Coffea liberica*** W.Bull, Nursery Cat. (William Bull) 97: 4 (1874).

*Global distribution:* west Tropical Africa (Benin, southern part), Ghana, Guinea, Ivory Coast, Liberia, eastern Sierra Leone, Nigeria); west-central Tropical Africa (Cabinda, Cameroon, Central African Republic, Congo, Democratic Republic of Congo, Gabon); north-east Tropical Africa (southern South Sudan); east Tropical Africa (Uganda); south Tropical Africa (Angola). Naturalised in Tropical Africa and other tropical areas (not listed here).

*Global IUCN conservation assessment:* Least Concern (LC). (Chadburn and Davis, 2017b).

**3a. *Coffea liberica*** var. ***liberica*** (not indigenous in Uganda).

*Global distribution:* west Tropical Africa (Benin, Ghana, Guinea, Ivory Coast, Liberia, Nigeria, eastern Sierra Leone); west-central Tropical Africa (Cabinda, Cameroon, Central African Republic, Congo, Democratic Republic of Congo, Gabon); south Tropical Africa (Angola). Naturalised in Tropical Africa and perhaps other tropical areas (not listed here); widely cultivated at small scale across the tropics.

*Global IUCN conservation assessment:* Not Evaluated.

**3b. *Coffea liberica*** var. ***dewevrei*** (De Wild. & T.Durand) Lebrun, Mém. Inst. Roy. Colon. Belge, Sect. Sci. Nat. (8vo) 11(3): 168 (1941).

*Global distribution:* west-central Tropical Africa (eastern Cameroon, Central African Republic, eastern Democratic Republic of Congo); north-east Tropical Africa (southern South Sudan); east Tropical Africa (western Uganda).

*Global IUCN conservation assessment:* Not Evaluated.

Represented in Uganda by the endemic taxon *C. liberica* var. *dewevrei* forma *bwambensis* (see note below).

**Figure 6:**
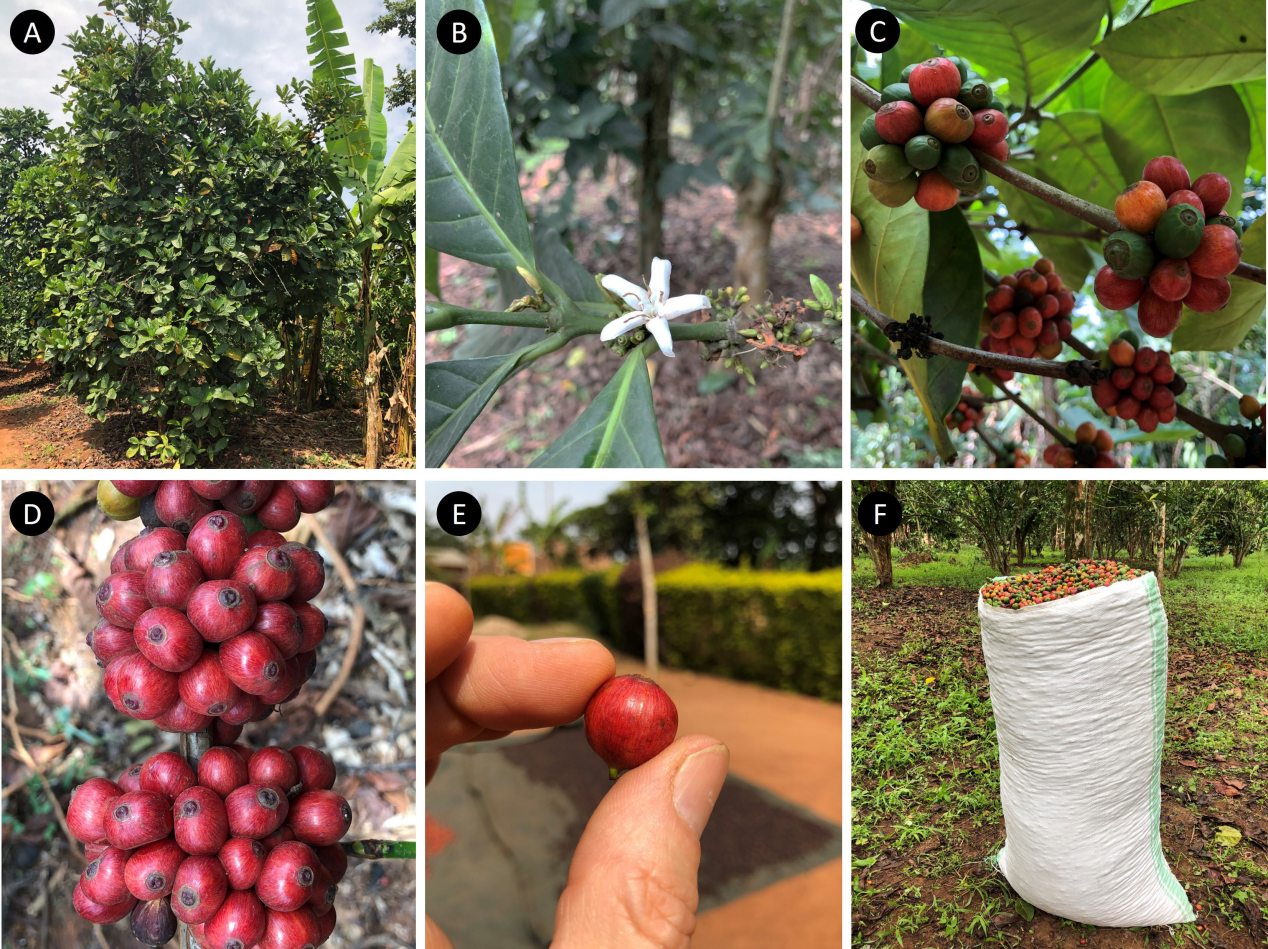
*Coffea liberica* var. *dewevrei* (excelsa coffee), cultivated in central Uganda. **(A)** Habit or farmed plant (tree), c. 5 m high, growing with banana; **(B)** Single flower (5-merous), old flowers and flower buds; **(C)** Fruiting branches, with maturing fruits; **(D)** Fruiting branch, with tight axillary clusters; **(E)** Single fruit (cherry), c. 17 × 15 mm; **(F)** sack of fruit (cherry) from a single tree (c. 70 kg).

**3b(i). *Coffea liberica*** var. ***dewevrei*** forma ***bwambensis*** Bridson, Kew Bull. 37: 314 (1982) **Figures 5A**, **6**.

***Information for Uganda***

*Distribution:* east Tropical Africa (western Uganda). Endemic to Uganda. Restricted to western Uganda, adjacent to the border with the Democratic Republic of Congo (in Semuliki Forest) and in north-eastern Uganda, in Zoka and at Kilak (Killak) Central Forest Reserves). Comprehensive fieldwork in Itwara Forest (during the years 2020 and 2021) shows that this species does not occur at this location, contrary to previous reports (Thomas, 1940b; Thomas, 1944; Kalema and Beentje, 2012; Kalema and Hamilton, 2020). **Figure 1C**.

*Ecology:* In medium elevation, humid, evergreen forest (Zoka Forest) and lowland semi-deciduous humid forest (Semuliki Forest), with a diverse range of dominant tree species and various forest communities. In Semuliki Forest, *C. liberica* var. *dewevrei* occurs mainly (c. 90%) in swamp forest, and even in places that support truly riverine species such as *Pandanus chiliocarpus* (screw pine), although it is not exclusively confined to these habitats in Semuliki Forest, and it grows in drier (soil) areas of this forest reserve that are not associated with water. In Zoka Forest, this species is predominantly found in areas that are not associated with rivers and waterlogged areas.

*Elevation:* 680*–*1200 m (observed and recorded); 686*–*1118 m (modelled).

*Preliminary country-level IUCN conservation assessment:* Endangered (EN). The Extent of Occurrence (EOO) is 7,716 km^2^ (Vulnerable (VU); and Area of Occurrence (AOO) is 64 km^2^ (Endangered (EN)). Whilst the EOO for *C. liberica* var. *dewevrei* in Uganda is substantial, a large part of the EOO is without populations of this species, and a substantial area includes Lake Albert (**Figure 1C**). Populations in Semuliki Forest (National Park) have a high density of individuals, and occur in a large proportion of the areas of the forest that have been surveyed (i.e. the eastern part). Encroachment and deforestation in Zoka Forest are affecting AOO, number of mature individuals, habitat quality and population health. Conservation management improvements are urgently required in Zoka Forest to ensure the Ugandan northernmost populations of this species are protected. Careful monitoring and management of this species *in situ* is urgently required for Uganda.

*Main protected area occurrence:* Semuliki (NP), Zoka (CFR). Key: Central Forest Reserve (FR), National Park (NP).

*Ugandan names:* From (Kalema and Hamilton, 2020): Mumwanyi (Luganda). Kisansa coffee (recorded on farms in the Luwero District).

*Other names: Coffea excelsa* A.Chev. (Botanical Latin; numerous authors). Excelsa coffee (English, numerous authors, widely used); Shari coffee [English; (Eggeling and Dale, 1952)], not widely used.

*Uses (other than beverage):* Not known.

*Notes:* The current consensus of taxonomic and systematic study (Lebrun, 1941; Bridson, 1988; N'Diaye et al., 2005; Davis et al., 2006; Baltazar and Buot, 2019; Panaligan et al., 2020; Panaligan et al., 2021) is that *C. liberica* should be divided into two botanical varieties: *C. liberica* var. *liberica* and *C. liberica* var. *dewevrei*. Whilst this view is generally accepted, it is also argued that the taxonomy of *C. liberica* does not fully account for the extreme morphological (Bridson, 1988; Stoffelen, 1998) and potential molecular variation (Charr et al., 2020) within the species, and thus requires further careful critical study. One of the main problems is that *C. liberica* has been introduced and become naturalised throughout tropical Africa, and so sampling for systematic studies may be biased. There also seems to be confusion around the plants identified as ‘excelsa’, a name that should only be used to refer to var. *dewevrei* but has been used, incorrectly, for variants of var. *liberica* (Davis et al., 2022). The botanical forma (f.) *bwambensis* (i.e. *C. liberica* var. *dewevrei* f. *bwambensis*) has been assigned to represent all indigenous Liberica coffee in Uganda, but the morphological circumscription may possibly also include populations in South Sudan (Bridson, 1988) and perhaps adjoining areas in the Democratic Republic of Congo. Given the uncertainty, in this contribution we refer to the Ugandan populations of Liberica coffee as *C. liberica* var. *dewevrei*.

In 1941, *C. liberica* var. *dewevrei* was found near Kilak (Killak), to the east of Zoka Forest, in riverine forest; fieldwork is required to ascertain whether this species still exists at this location.

**4. *Coffea neoleroyi*** A.P.Davis, Phytotaxa 10: 43 (2010).

*Global distribution:* north-east Tropical Africa (south-western Ethiopia, and south-western South Sudan); east Tropical Africa (north-eastern Uganda).

*Global IUCN conservation assessment:* Endangered (EN) (O'Sullivan et al., 2017).

***Information for Uganda***

*Distribution:* Restricted to Mt. Zulia in north-eastern Uganda **Figure 1D**.

*Ecology:* On riverbanks, and in seasonally dry *Combretum*-*Terminalia* savanna woodland, often amongst boulders.

*Elevation:* c. 1200 m.

*Preliminary country-level IUCN conservation assessment:* Critically Endangered (CR) or Data Deficient (DD). The Extent of Occurrence (EOO) cannot be calculated owing to a single data point; Area of Occurrence (AOO) is 4 km^2^ (CR), based on a single grid cell with a cell width of 2 km. Realistically, at the country-level, this would be better placed in the DD category, given that there is only a single collection, and the area where this species grows is isolated, and has not been the subject of detailed botanical survey; dedicated survey work for this species in north-eastern Uganda is urgently required.

*Protected area occurrence:* Zulia (FR). Key: Forest Reserve (FR).

*Ugandan names:* Not known.

*Other names:* Not known.

*Uses (other than beverage):* Not known.

*Notes:* A rare and untypical *Coffea* species, formerly included in *Psilanthus* (Davis et al., 2011), which is characterised by its deciduous habit, and long-tubed flowers (corolla) with very short (included) styles (Davis et al., 2005). All other Ugandan *Coffea* species and most (but by no means all) other *Coffea* species have evergreen leaves, short-tubed flowers, and a long (excluded) style. Like all *Coffea* species, *C. neoleroyi* produces a fruit that contains two seeds, and each seed possesses the typical coffee-bean morphology. The seeds (coffee beans) of *C. neoleroyi* are much smaller than *C. canephora* and *C. liberica*, and smaller than *C. eugenioides* (**Figure 5**).

### Prior and existing uses of wild coffee resources within the Ugandan coffee sector

#### *Coffea canephora* (robusta coffee)

The commercial use of *C. canephora* in Uganda dates to at least the mid-1800s, when various observers recorded farming for local use (as a product for chewing and consumption, rather than as a beverage), and national cross-border trade (Thomas, 1935; Thomas, 1940a; Wrigley, 1988; Kiwuka et al., 2021). Traditional and ritualistic uses of this species in Uganda are long-established, although the historical time-line is unclear (Thomas, 1935; Thomas, 1940a). Arabica coffee (*C. arabica*) was believed to have been introduced into cultivation in Uganda in 1900 (Thomas, 1940c), at which time coffee cultivation in general (including *C. canephora*) had started to be promoted as a key agricultural export, gathering increased momentum from the 1910s onwards (Thomas, 1940a).

A recent survey of the genetic diversity of *C. canephora* (Kiwuka et al., 2021) using microsatellite (SSR) markers on a comprehensive sampling of wild and cultivated accessions from Uganda, as well as indigenous populations of this species across Africa, has provided considerable enlightenment regarding the origin of farmed *C. canephora* in Uganda. The analyses of Kiwuka et al. (2021) infer that indigenous populations from the forests of Malabigambo, Mabira, and Kalangala (Ssese Islands), i.e. the southern-central (SC) genetic cluster of Kiwuka et al. (2021), as well as introduced germplasm from other parts of Africa, represent the origin of the farmed robusta used today in Uganda. There is thus agreement with what is known about the early years of coffee cultivation in Uganda, particularly with reference to the development of robusta coffee on the Ssese Islands and surrounding areas on the mainland (Thomas, 1940a; Thomas, 1944; Wrigley, 1988). The study confirms that plants of Congolese origin (i.e. Democratic Republic of Congo) were introduced into Uganda as part of the effort to upscale coffee production in Uganda, even though there were plentiful indigenous *C. canephora* resources present in the forests of Uganda: the transfer of western African *C. canephora* to Uganda is recorded in the literature (Cheney, 1925; Thomas, 1935). The movement of *C. canephora* from other parts of Africa, may have been direct, or indirect, for example *via* Java (Thomas, 1935; Wrigley, 1988). The probable reasons for the introduction of non-Ugandan germplasm were that *C. robusta* (now a synonym of *C. canephora*) was then considered a separate species to *C. canephora*, and that the variation across the two species included specific positive traits and qualities (Cheney, 1925; Thomas, 1935) and would have thus been considered as worthy introductions. It has been suggested (Thomas, 1935) that the Nganda type of *C. canephora* (which has a spreading habit) may have originated from a forest in Uganda, whereas the Erecta type (upright habit) was introduced from the Congo Basin. In their analyses Kiwuka et al. (2021) these morphological types are, however, intermixed with individuals of the SC group, of both non-Uganda and Ugandan origin, indicating that their phenotypic differences are not clearly distinguishable (at least, on the basis of the 19 microsatellite (SSR) markers used), or may have no genetic foundation. There may have been a number of man-made introductions from a range of countries/populations within Africa, although in some cases the genetic similarity may be signalling natural relationships (i.e. without human intervention) with contiguous and perhaps non-contiguous regions across tropical Africa (Kiwuka et al., 2021). In summary, and based on the evidence at hand, it is inferred that most of the farmed germplasm of Uganda originated from the southern-central forests [the SC cluster of Kiwuka et al. (2021)], including Malabigambo, Kalangala, Mabira, but with admixture of material from other countries, and perhaps intermixing (via spontaneous crosses) between indigenous and introduced genotypes; although the relative contributions of these three factors would be difficult to assess (Kiwuka et al., 2021). The results of Kiwuka et al. (2021) are consistent with the claim that in the latter part of 1800s, plantation owners and smallholder farmers in the Lake Victoria Basin region began cultivating *C*. *canephora* using directly sourced wild coffee (Thomas, 1935; Thomas, 1940a; Thomas, 1944), although there would have been a considerable amount of selection for the best performing variants at that time (Thomas, 1935). Kiwuka et al. (2021) also revealed that all of the six elite clones (KW13, KW14, KW15, KW16, KW18 and KW19) possessing coffee wilt disease (CWD) resistance and high yield characteristics (Mulindwa et al., 2022), and which have provided the mainstay of modern robusta production in Uganda, are genetically similar to wild SC populations (Kiwuka et al., 2021). It should also be said that a large proportion of globally cultivated robusta originated from Uganda, including those grown by major coffee producers, such as Vietnam and Mexico (Garavito et al., 2016). Kiwuka et al. (2021) also show that the genetic diversity found in Uganda’s north-western forests (Zoka, Budongo, Itwara and Kibale) is distinct from the germplasm currently employed in Uganda’s coffee farming sector. This is noteworthy because these populations occur in comparatively warmer and drier climatic zones, and may have climate resiliency attributes (Kiwuka et al., 2021), i.e. higher thresholds to abiotic stressors, such as higher temperatures and lower soil moisture.

### Coffea eugenioides

Early trials of *C. eugenioides* as a beverage species, in East Africa (including Uganda), were unfavourable due to the small size of its seeds (coffee beans) (Thomas, 1944), susceptibility to coffee leaf rust (Bullock, 1930), poor quality, and very small yields (Thomas, 1940b; Thomas, 1944). Early sensory assessments, were not unfavourable, however, for example: “The liquor was described as pure and entirely free from undesirable flavours, although the strength was not good, probably owing to the presence of immature trees” (Thomas, 1940b); and …“the quality of the bean is mild and agreeable”… (Thomas, 1944). Outside Uganda, and more recently, the flavour of *C. eugenioides* has received praise, for example: “The exception is *C. eugenioides*, which has a very fine aroma, tasting fruity and clean.” (Fazouli et al., 2000). Trial plantings of *C. eugenioides* were made in Uganda during the 1920s and 1930s (as evidenced by samples housed at the Economic Botany Collection, RBG Kew), but no further development of the species seems to have been undertaken there. In south-eastern Kenya, small scale production of *C. eugenioides* has been underway for several decades, as Nandi coffee, although the current status of this crop is unclear. In Colombia, *C. eugenioides* is presently grown on a small scale (i.e. on a single estate that produces specialty (high quality) coffee), which sells at a substantial premium, and has been used in national and international coffee making competitions (i.e. the World Barista Championships) on account of its unique, complex flavour and intense natural sweetness. *Coffea eugenioides* is reported to be difficult coffee to grow, and low yielding (e.g. 150 grams per tree of un-milled coffee; https://cafeinmaculada.com/en/blogs/varieties/variedades). Based on the renewed interest, and high market price, preliminary trials of *C. eugenioides* are now underway in Uganda.

*Coffea eugenioides* has been used as a breeding partner, for imparting flavour qualities and other attributes *via* crosses with other species. Spontaneous diploid (2n=22) crosses between *C. eugenioides* and *C. liberica* have been identified (Maurin et al., 2007) and artificial tetraploid crosses (‘Ligenioides’) have been made (Reddy et al., 1985; Ganesh et al., 2002), and evaluated (see below). Tetraploid (2n=44) versions of this interspecies hybrid can be readily backcrossed with *C. arabica* (2n=44) and *C. canephora* (using the Timor Hybrid) to produce hybrids with the potential for commercial use (Ganesh et al., 2002). Diploid, artificial hybrids between *C. eugenioides* and *C. canephora* have also been made, which after chromone doubling (2n=44) crossing with *C. arabica* and then backcrossing, have produced a line of high yielding hybrids with acceptable beverage quality and high productivity (Nagai et al., 2008).

### *Coffea liberica* var. *dewevrei* (excelsa coffee)

*Coffea excelsa* A.Chev. (Chevalier, 1903) and *C. dewevrei* De Wild. & Th. Dur. (Durand and De Wildeman, 1899) are synonyms of *C. liberica* var. *dewevrei* (Davis et al., 2006). *Coffea excelsa* features predominately in early references of this wild plant in Uganda (Thomas, 1940b; Tothill, 1940; Thomas, 1944), and elsewhere (Cheney, 1925; Wellman, 1961; Wrigley, 1988). Indeed, the common name ‘excelsa’ is frequently applied to this plant, just as Arabica and robusta are applied to *C. arabica* and *C. canephora*. It should be noted, however, that the name ‘excelsa’ whether used as a common name, or as a species epithet, is often incorrectly applied to small to medium sized seed (coffee bean) variants of *C. liberica* var. *liberica* (Davis et al., 2022), particularly those cultivated in Asia but also parts of Africa. The common name ‘excelsa’ should be restricted to those plants conforming to *C. liberica* var. *dewevrei* (Bridson, 1988; Davis et al., 2022) originating from the eastern Cameroon, Central African Republic, eastern Democratic Republic of Congo and western Uganda.

Field trials of excelsa coffee in in Uganda, during 1915 and 1916, indicated poor yields (Cheney, 1925; Thomas, 1940b). However, the material used was imported from Java in 1914, and is certainly not *C. liberica* var. *dewevrei* (excelsa) but rather *C. liberica* var. *liberica*, as indicated by the large size of its seeds (Davis et al., 2022). In other countries, cultivated material of *C. liberica* var. *dewevrei* (as *C. excelsa*) was more thoroughly assessed and received favourable reviews. Many identified considerable potential for excelsa as a coffee crop species (Freeman and Chandler, 1907; Cramer, 1913; Cheney, 1925; Chevalier, 1929; Cramer, 1957). For example [translated from French]: “Many farmers consider it to have a great future, as it is very resistant to diseases and insects, and it gives high yields of good quality coffee’ (Chevalier, 1929). Some of the information pertaining to excelsa coffee is likely to be misplaced owing to the confusion between var. *liberica* (Liberica/Liberian coffee) and var. *dewevrei* (excelsa) (Davis et al., 2022), as indicated above.

Over recent decades, the timing of which is not clear but which may date back to at least the 1980s, there has been a dramatic increase in the number of farmers in Uganda (perhaps more than 200) growing *C. liberica* var. *dewevrei*, either with *C. canephora*, or as the dominant coffee crop (Davis et al., 2022). The shift to *C. liberica* var. *dewevrei* has been farmer-led, and has occurred independently of extrernal influences, other than minor interest in purchasing for export as a differentiated coffee (Davis et al., 2022). According to the farmers in lowland Uganda growing *C. liberica* var. *dewevrei*, this plant has been on their farms, in low numbers, for many decades, and was originally gathered from the forest by previous generations (Davis et al., 2022), although this requires confirmation. Preference for farming *C. liberica* var. *dewevrei* over *C. canephora* appears to be the result of production issues with *C. canephora* (robusta), and particularly the increasing occurence and severity of disease (especially coffee wilt disease), pests (particularly stem/twig borers) and droughts. A similar upsacling of *C. liberica* var. *dewevrei* is also occurring in South Sudan (Davis et al., 2022). Farmers in Uganda consistently report high yields for *C. liberica* var. *dewevrei*, which based on yield-per-plant of fresh fruit (e.g. **Figure 6F**) and an outturn (conversion) ratio (kg of fresh fruit: kg clean coffee) of 7:1, ranges between 877 kg/ha (204 trees/ha) to 3,440 kg/ha (400 trees/ha), for rain-fed, low input (e.g. negligible fertiliser use) farming systems (Davis et al., 2022). As yet, there are no reports of coffee wilt disease (Davis et al., 2022), which is a widespread and devastating disease of *C. canephora* in Uganda. Improved monitoring and further research is required to asess the level of resistance of coffee wilt disease in *C. liberica* var. *dewevrei*, as it was first reported on this species in the Central African Republic in 1927, and later caused widespread damage to Liberica and robusta coffee across large areas of tropical Africa (Gaitán et al., 2015).

Unlike *C. arabica* and *C. canephora*, *C. liberica* var. *dewevrei* grows into a substantial, medium sized tree (**Figure 6A**), of around 10 m or more. In Uganda, the fruit development period of *C. liberica* var. *dewevrei* is longer than the aforementioned crop species, and the main harvest periods do not overlap: the harvest period for *C. liberica* var. *dewevrei* is January to April. The fruits of *C. liberica* var. *dewevrei* are held in tight axillary clusters (**Figures 6C, D**), like *C. canephora* and many cultivars of *C. arabica*, but unlike many variants of *C. liberica* var. *liberica.* The fruits and seeds of *C. liberica* var. *dewevrei* are approximately the same size and dimensions (**Figures 6C–E**) as *C. arabica* (**Figure 5E**). This presents a distinct advantage over the large-fruited, thick pulped variants of *C. liberica* var. *liberica*, as processing can be carried using standard procedures and the outturn (conversion ratio of fresh fruit to clean coffee) is much more satisfactory (Davis et al., 2022).

*Coffea liberica* var. *dewevrei* (excelsa coffee) produced in Uganda and South Sudan yields a coffee that is smooth and easy-drinking, with low to medium acidity, low bitterness, possessing a range of positive flavour notes, and a caffeine content similar to *C. arabica* (Davis et al., 2022).

In Uganda, *C. liberica* var. *dewevrei* is mostly exported as, or mixed with, *C. canephora* (robusta). This is partly due to confusion over the identity of excelsa (sometimes considered a large, thick-leaved type of robusta) and because of convenience. A separate market for excelsa coffee (*C. liberica* var. *dewevrei*) does not exist in Uganda, although there have been limited exports of this coffee to Italy in recent years, as Kisansa coffee (https://www.fondazioneslowfood.com/en/ark-of-taste-slow-food/kisansa-coffee/) and in 2022 to the UK (Clifton Coffee, personal communication).

*Coffea liberica* var. *dewevrei* may also have considerable utility in the Ugandan coffee sector as grafting stock. *Coffea liberica* sensu lato is used to improve resistance to root nematodes, and increase yield and survivability of grafted *C. arabica*, particularly in Hawaii (Myers et al., 2020). In Uganda, *C. liberica* var. *dewevrei* could be used for grafting of CWD resistant *C. canephora* clones. Currently, CWD resistant *C. canephora* is reproduced by cuttings, which although successful means that a tap root is not formed. Plants (scions) of *C. canephora* would likely benefit from the stout tap root and extensive, robust root system of *C. liberica* var. *dewevrei*, particularly under low soil moisture conditions. Further research is warranted, including the identification of the most suitable grafting stock, as undertaken for the grafting of *C. arabica* onto *C. liberica* var. *liberica* stock in Hawaii (Myers et al., 2020).

*Coffea liberica* var. *liberica* has been used in coffee crop development, *via* hybridization with other beverage species. It has contributed coffee leaf rust resistance to the widely grown Indian cultivar *C. arabica* ‘S.795’, *via* the progenitor cultivars *C. arabica* ‘S.288’ and ‘S.26’ (Narasimhaswamy, 1960; Surya Prakash et al., 2002). In Indonesia, crosses between *C. liberica* var. *liberica* and *C. arabica* have provided a number of tetraploid (and octaploid) hybrids, most notably the ‘Kalimas’ and ‘Kawisari’ hybrids, which also have a high degree of resistance to coffee leaf rust, and in some cases high yields and a fair market price (Cramer, 1957). Diploid hybrids between *C. canephora* and *C. liberica* (possibly var. *dewevrei*) have also been documented (Cramer, 1957; Chinnappa, 1970). As mentioned above (for *C. eugenioides*), *C. liberica* hybridizes with *C. eugenioides*, to form diploid and tetraploid hybrids. In India, these hybrids showed good yield potential and coffee leaf rust resistance, although the need for further development was identified (Reddy et al., 1985; Ganesh et al., 2002). In Madagascar, tetraploid *C. liberica* and *C. eugenioides* have been crossed, and then backcrossed with *C. arabica*, to produce high-yielding hybrids with acceptable sensory characteristics, as part of the ‘Ratelo Hybrid’ programme (Jean Jacques Rakotomalala, personal communication). Thus, in Uganda, indigenous *C. liberica* var. *dewevrei* could offer potential as a breeding partner, for imparting required traits to new coffee crop plants, *via* interspecies crosses.

### Coffea neoleroyi

Several factors preclude *C. neoleroyi* from being a coffee crop species, including extremely low yields (due to the production of low numbers of fruits per tree, and small seed (coffee bean) size, diminutive stature and spindly growth form. It is possible that a coffee-like beverage could be made from the seeds of *C. neoleroyi*, but this remains untested. Given the differences in floral morphology between *C. neoleroyi* and coffee beverage crop species (Davis et al., 2005) breeding with other coffee species would be difficult (Couturon et al., 1998). Due to the rarity and geographical isolation of *C. neoleroyi* in Uganda, and indeed in the other known locations for this species (South Sudan and Ethiopia), this species remains poorly known.

### Climate profiling

Our basic modelled climate data analysis for the four wild coffee species of Uganda is summarised in **Table 1**. A scatter plot of annual mean temperature (Bio1) vs. total mean annual precipitation (Bio12) for Ugandan coffee species is given in **Figure 2**; *C. neoleroyi* is included for illustrative purposes only (as there is only a single data point). Density plots for Bio1, Bio12, precipitation seasonality (Bio15), and elevation are given in **Figure 3** (except for *C. neoleroyi*). The modelled mean annual temperatures, annual precipitation and precipitation seasonality for the Ugandan populations are: *C. canephora* (21.8°C/1389 mm/44), *C. eugenioides* (21.3°C/1370 mm/42), and *C. liberica* var. *dewevrei* (24.4°C/1560 mm/51). These data (**Table 1**; **Figures 2**, **3**) show that *C. canephora*, *C. eugenioides* and *C. liberica* var. *dewevrei* overlap for Bio1, Bio12 and Bio15, which is not surprising given that these species overlap in their distributions (**Figure 1**), especially *C. canephora* and *C. eugenioides*. A greater density of warmer mean annual temperature (Bio1) for *C. liberica* var. *dewevrei* (compared to *C. canephora* and *C. eugenioides*) is evident because this species is confined to lower elevation forests in western and northern Uganda (**Figures 1**, **2**). *Coffea liberica* var. *dewevrei* has a higher density of occurrences in locations with a higher total mean annual precipitation (Bio12), and precipitation seasonality (Bio15), as shown in **Figure 3**. The higher density of lower precipitation (Bio1) and lower precipitation seasonality (Bio15) for *C. canephora* and *C. eugenioides* is due to the higher number of datapoints in drier locations, compared to wetter locations, biased by the higher number of datapoints for these species overall. For *C. liberica* var. *dewevrei*, the density distribution for Bio12 (i.e. number of wetter locations) is bimodal (**Figure 3A**) due to the disparity in rainfall for the north-western locations (Zoka Forest and Kilak (Killak); 938–1580 mm per year), vs. those in central-western (Semuliki National Park; c. 2200 mm), and because the number of ground-point data records are the same in each area (five). The values for precipitation seasonality (Bio 15) are higher in *C. liberica* var. *dewevrei* (51), compared to *C. canephora* and *C. eugenioides* (44 and 42), but given the above-stated considerations (including the low number of samples) any firm interpretations are inadvisable. The global values (i.e. across the entire indigenous distribution) for *C. canephora* are higher [56; (Davis et al., 2021b)] than for Uganda alone (44). Given the higher precision expected with a national (Uganda only) rather than global (across the African continent) the lower figure is likely to be more meaningful.

**Table 1:** Basic climate profiling and elevations for three indigenous Ugandan coffee species, based on location records for Ugandan distributions, with comparative data from published sources.

Species	Min./max. mean values (data points); and mean of all data points (bold)	Mean annual temperature (°C)	Mean total annual precipitation (mm/year)	Precipitation seasonality	Elevation (m)
		CHELSA Bio1	CHELSA Bio12	CHELSA Bio15	
*C. canephora*	Min.	18.7	851	31	675
	Max.	24.7	2144	54	1666
	**Mean**	**21.8**	**1389**	**44**	**1187**
*C. eugenioides*	Min.	18.7	857	31	700
	Max	24.6	2544	54	1693
	**Mean**	**21.3**	**1370**	**42**	**1261**
*C. liberica* var. *dewevrei*	Min.	23.5	983	46	686
	Max	25.2	2115	58	1118
	**Mean**	**24.4**	**1560**	**51**	**857**
Published mean values for annual temperature and precipitation (Davis et al., 2021b); and precipitation seasonality (Davis et al., 2021a).					
*C. arabica*		**18.7**	**1614**	**58**	
*C. canephora*		**23.7**	**1601**	**56**	
*C. liberica* sensu lato		**23.9**	**1699**		

Mean minimum, mean maximum and mean annual temperatures (CHELSA Bio1); mean total annual rainfall (CHELSA Bio12), and precipitation seasonality (CHELSA Bio15), from the CHELSA modelled dataset (Karger et al., 2017). Numbers in bold indicate mean of all data values. Data for *C. neoleroyi* not given (single datapoint only).

## Discussion

### Survey of wild coffee species and conservation priorities

There are four indigenous (wild) coffee species in Uganda: *C. canephora*, *C. eugenioides*, *C. liberica* var. *dewevrei* and *C. neoleroyi*. *Coffea canephora* and *C. eugenioides* are widespread in western Uganda (**Figures 1A, B**) where suitable forest habitat exists, although in central Uganda many populations occur in small and often degraded forest parcels, which require improved safeguarding. Uganda represents important centres of diversity for *C. canephora*, *C. eugenioides* and *C. liberica* var. *dewevrei*, and all three are priority species for coffee crop plant development (i.e. Coffee Crop Wild Relative (CWR) Group 1; (Davis et al., 2019). *Coffea liberica* var. *dewevrei* is restricted to three populations in western Uganda (**Figure 1C**). In Semuliki Forest (National Park) the population appears to be quite extensive, healthy, and with a reasonably high density individuals. The forested area covers most of the Semuliki protected area boundary (219 km²). Conversely, in Zoka Forest the population is under threat from encroachment and is chronically suffering from reduced forest cover and poor forest health. The third population, at Kilak (Killak), has not been surveyed for *C. liberica* var. *dewevrei* since it was last recorded there (Thomas, 1944); dedicated fieldwork in this area is required. All of the records used by Thomas (1940b); Thomas (1942); Thomas (1944) for his surveys of indigenous coffee species were based on, or vouchered, using herbarium specimens, except the records used for *C. liberica* var. *dewevrei* at Itwara, which were based on observation only. It is likely that he, or his informants, mistakenly identified large-leaved variants of *C. canephora* (which the authors have seen in Itwara) as *C. liberica* var. *dewevrei*. It could be argued that the *C. liberica* var. *dewevrei* once occurred in this forest and has since been extirpated due to partial deforestation, but this seems unlikely. There are plentiful herbarium specimens for *C. canephora* and *C. eugenioides* collected from Itwara, and it seems likely that at least one specimen of *C. liberica* var. *dewevrei* would have been collected, especially given the relatively easy access to this forest. *Coffea neoleroyi* is only known from a single collection (**Figure 1D**) in a remote area of north-eastern Uganda. Further dedicated field survey for this species is required, to fully understand the number and density of populations in Uganda, and across its natural range (i.e. Uganda, Ethiopia and South Sudan).

Our preliminary country-level IUCN Red List conservation assessments (IUCN Standards and Petitions Subcommittee, 2022) for the four indigenous coffee species of Uganda are: *C. canephora* (Least Concern), *C. eugenioides* (Least Concern), *C. liberica* var. *dewevrei* (Endangered), and *C. neoleroyi* (Critically Endangered, or Data Deficient). Under a Least Concern rating, individual populations may still be at risk of extirpation, as is the case for *C. canephora* and *C. eugenioides*. In terms of genetic resources, and their value to the Ugandan coffee sector, this review shows that potentially useful attributes (diseases resistance, climate resiliency, etc.) are distributed across populations (as well as the species as a whole) and thus require conservation. The rating of Endangered for *C. liberica* var. *dewevrei* is of considerable concern, particularly given the level of forest clearance and land use change at Zoka Forest. Further data is required before a confident extinction assessment can be made for *C. neoleroyi*, but this is also undoubtedly also a species of concern.

### Climate profiling

In Uganda, indigenous *C. canephora*, *C. eugenioides* and *C. liberica* var. *dewevrei* occur (grow and reproduce) over the same range of basic climate variables (Bio1, mean annual temperature; Bio12, mean annual precipitation; and Bio15, precipitation seasonality) as summarised in **Table 1** and **Figures 2**, **3**. *Coffea liberica* var. *dewevrei* occurs in much warmer locations (mean annual temperature 24.4°C) than *C. canephora*, *C. eugenioides* (21.8°C and 21.3°C, respectively) on account of it being restricted to low elevation forests. The elevation restriction may not be due to inability, or lack of opportunity, for this species to exist at higher elevations, although there could be intrinsic factors in play. There is also the possibility that the distribution and elevation range of *C. liberica* var. *dewevrei* in Uganda may have been more extensive historically, prior to forest clearance by humankind. The bimodality in mean precipitation (Bio12) and higher precipitation seasonality (Bio 15) for *C. liberica* var. *dewevrei* (compared to *C. canephora* and *C. eugenioides*; **Figure 3**) may infer further climatic differences (other than a mean temperature difference) but based on the data at hand no firm assumptions can be made. Field observations made by us show that *C. liberica* var. *dewevrei* is often associated with high water tables. It occurs in swamp forest (at Semuliki National Park) and can be close to rivers (Zoka Forest), and outside Uganda it has been often recorded in gallery forest in native habitats (Chevalier, 1929). However, it is by no means exclusive to these wetter habitats, as observed by us in Zoka Forest. Published observations (Thomas, 1940b; Thomas, 1944) and farmer feedback (from farm observation and farmer feedback during dry spells in 2021 and 2022) indicate that *C. liberica* var. *dewevrei* is more drought tolerant than *C. canephora*. Drought tolerance assumptions and observations for *C. liberica* var. *dewevrei* (as *C. excelsa*) have been made by other workers (Anon, 1890; Cheney, 1925; Cramer, 1957). The modelling approach used here neither supports nor refutes drought tolerance (e.g. higher precipitation seasonality), particularly as the Bio15 values for *C. canephora* (precipitation seasonality (PS) value = 44) *C. liberica* var. *dewevrei* (PS = 51) are not that far apart (**Table 1**; **Figure 3E**). However, the values for indigenous *C. arabica* (Ethiopia and South Sudan (PS = 58; (Davis et al., 2021b); and indigenous Ugandan *C. canephora* (PS = 44) infer that there are precipitation seasonality differences between these two species. This is supported by observation of wild and farmed populations of *C. canephora*, which generally occur in locations with lower precipitation seasonality than *C. arabica* (A. Davis personal observation), although these relationships are complex (and probably fine-scaled) and require careful evaluation. *Coffea racemosa* and *C. zanguebariae*, two species occurring in areas of extremely seasonal rainfall, can have Bio15 values of 90 (Davis et al., 2021a), indicating the scale of difference in precipitation seasonality between *Coffea* species. Multi-location variety trials (MLVTs) are required to substantiate the analyses and observations presented here, and better understand the climatic tolerances of indigenous Ugandan coffee species, and cultivated Arabica coffee. Experimentation of this nature would be critical for understanding the value of Uganda’s wild species resources for crop development, across different agroecological conditions under a changing climate.

### Prior, existing and future uses of wild coffee resources

Molecular analyses (Kiwuka et al., 2021) have substantiated the assumption that indigenous populations of *C. canephora* (robusta coffee) have provided [after selection (Thomas, 1944)] Uganda with the bulk of the germplasm on which their robusta farming sector is based (Thomas, 1940a; Thomas, 1944), as elaborated above (see Results). Indigenous coffee natural capital has also provided the resources for sustaining robusta production in Uganda and other countries (Garavito et al., 2016). In particular, wild germplasm (Kiwuka et al., 2021) has played a key role in developing cultivars (several clones) to combat the devastating effects of coffee wilt disease (Rutherford, 2006; Musoli et al., 2009; Musoli et al., 2013; Mulindwa et al., 2022), which ravaged robusta production in Uganda in the 1990s and remains an ongoing issue. Despite the considerable contribution already made by using indigenous *C. canephora* resources, much remains untapped (Ngugi and Aluka, 2019; Kiwuka et al., 2021) and may prove to be of value for addressing sustainability issues. For example: germplasm from wild populations of *C. canephora* may offer climate resiliency potential, particularly those from warmer and drier forests, such as Zoka Forest (Kiwuka et al., 2021); it has been shown that wild collections of wild robusta from Kalangala and Itwara forests have a high level of resistance to coffee wilt disease (Phiri and Baker, 2009); and selections from Itwara have shown promise under plantation conditions (Thomas, 1944).

The recent farming uptake of *C. liberica* var. *dewevrei* (excelsa coffee) represents an interesting development, and may offer considerable potential, as a third coffee crop species for Uganda (after *C. arabica* and *C. canephora*). Excelsa coffee fulfils farmers requirements, as it is easy to grow, appears to possess resistance to many of the major pests and diseases of coffee, is high yielding, and has an acceptable conversion (outturn) ratio from fresh fruit to clean coffee, particularly compared to the large fruited, thickly pulped types of *C. liberica* var. *liberica* (Davis et al., 2022). *Coffea liberica* var. *dewevrei* may be more tolerant of higher temperatures, compared to *C. canephora* in Uganda (mean annual temperature 24.4°C vs. 21.8°C; **Table 1**), but this requires careful assessment *via* field trials. Farmers in Uganda have reported better performance (personal communication) of *C. liberica* var. *dewevrei* over *C. canephora* during drought conditions (in 2021 and 2022). *Coffea liberica* var. *dewevrei* is certainly more heat tolerant than wild and cultivated *C. arabica*, which has a mean annual temperature range of 18–22 °C (Alègre, 1959; DaMatta and Ramalho, 2006; Davis et al., 2021b). It is clear that *C. arabica* cannot be grown successfully alongside *C. liberica* var. *dewevrei* in most lowland conditions in Uganda, even though it may persist over the short-term (Thomas, 1940c; Haarer, 1962). Whilst our climate analyses are not in conflict with these observations they do not support them with any degree of confidence. The thick, fleshy leaves, stout drunk and extensive root system of *C. liberica* var. *dewevrei* are features that are likely to constitute drought (and heat) tolerance advantages over *C. canephora*. Under wet soil conditions in Uganda, the root-system of *C. canephora* has been reported to be superficial and shallow (Thomas, 1944), which is likely to make this species susceptible during drought periods. It has also been noted that *C. liberica* var. *dewevrei* is tolerant of a wide range of soils (Thomas, 1944). *Coffea liberica* var. *dewevrei* (excelsa) is capable of producing economically viable, good quality coffee (see above) but further assessment is required to see how it will perform across the value chain. This species may also offer useful prospects as a rootstock for *C. canephora* and perhaps other coffee crop species, and for coffee crop development *via* breeding (see Results for the potential of both uses).

Due to its highly desirable flavour qualities, *C. eugenioides* may offer potential as a niche crop for the high-value sector of the coffee market, as it has in Central America. The development of *C. eugenioides* in Uganda would require investment and a proof-of-concept period, to test for commercial viability. The small seed (coffee bean) size and low yields represent key constraints, unless better performing variants can be found within wild populations, although this seems unlikely based on the field surveys we have carried out so far. *Coffea eugenioides* is likely to offer better potential as a breeding partner, for imparting flavour qualities and other attributes *via* interspecies crosses.

The wild diversity of *C. eugenioides* and *C. canephora* in Uganda could be of paramount interest, since they are the progenitors of *C. arabica* and could be used to produce Arabica analogues. Moreover, it has been shown that the *C. canephora*-derived sub-genome of *C. arabica* is closely related to the *C. canephora* accessions from northern Uganda, and in particular Zoka Forest (Merot-L'anthoene et al., 2019).

## Conclusion

In this contribution we enumerate four indigenous coffee species for Uganda (*C. canephora*, *C. eugenioides*, *C. liberica* (var. *dewevrei*) and *C. neoleroyi*) and provide new ecogeographical data summaries (and other information) for each of these species. Climate profiling, *via* simple modelling methods, shows overlap for basic climate requirements for three of these species (*C. neoleroyi* was excluded due to lack of data), although *C. liberica* var. *dewevrei* has a higher density of individual records in locations of higher temperature, and a higher precipitation seasonality. At the national level, a draft IUCN Red List assessment indicates that *C. liberica* var. *dewevrei* is Endangered, and that *C. neoleroyi* could be Critically Endangered. Many wild *Coffea* populations in Uganda are compromised due to land use change (e.g. deforestation and agricultural encroachment) and some populations may be threatened with extirpation. The considerable indigenous diversity reported for *C. canephora*, and assumed diversity for *C. eugenioides* and *C. liberica* var. *dewevrei*, based on number of populations and range, represents valuable natural capital for crop development (e.g. *via* breeding) and the sustainability of the Uganda coffee sector in general, particularly under changing climatic conditions. Wild populations of *C. canephora* have provided Uganda with the bulk of diversity for the establishment and sustainability of its thriving robusta coffee sector. *Coffea liberica* var. *dewevrei* (excelsa coffee) shows potential as stand-alone crop species, and as a source of grafting stock for *C. canephora* (robusta coffee) and other coffee species. The coffee natural capital of Uganda requires improved protection, in order to avoid the loss of genetic diversity and coffee crop development options.

## Data availability statement

The original contributions presented in the study are included in the article/supplementary materials. Further inquiries can be directed to the corresponding author.

## Author contributions

AD designed the research project, undertook the research and some of the analyses, and wrote the paper; CK designed the research project and undertook the research; AF designed the research, undertook the research and most of the analyses; JM provided ideas, guidance and logistic support; JK designed the research project and undertook the research. All authors contributed to the article and approved the submitted version.

## References

[B1] Alègre C. (1959). Climates et caféiers d´Arabie. Agron. Trop. 14, 23–58.

[B2] Anon (1890). Liberian Coffee. Bull. Misc. Inform. (Royal Bot. Gard. Kew). 47, 245–253.

[B3] Avelino J. Cristancho M. Georgiou S. Imbach P. Aguilar L. Bornemann G.. (2015). The coffee rust crises in Colombia and central America, (2008–2013): impacts, plausible causes and proposed solutions. Food Secur. 7, 303–321. doi: 10.1007/s12571-015-0446-9

[B4] Bachman S. Moat J. Hill A. de Torre J. Scott B. (2011). Supporting red list threat assessments with GeoCAT: geospatial conservation assessment tool. ZooKeys. 126, 117–126. doi: 10.3897/zookeys.150.2109 PMC3234434 22207809

[B5] Baltazar M. D. Buot I. E. (2019). Leaf architectural analysis of taxonomic confusing coffee species: *Coffea liberica* and *Coffea liberica* var. *dewevrei* . Biodiversitas. 20, 1560–1567. doi: 10.13057/biodiv/d200611

[B6] Bharatha Nandhini R. M. Rahul R. N. Thilaga S. Surya Prakash Rao N. Ganesh D. (2013). Molecular distinction of C×R hybrid (*Coffea congensis*×*Coffea canephora*) from morphologically resembling male parent using rbcL and matK gene sequences. S. Afr. J. Bot. 88, 334–340. doi: 10.1016/j.sajb.2013.08.011

[B7] Bridson D. M. (1988). “ *Coffea*,” in Flora of Tropical East Africa, Rubiaceae, part 2. Eds. Polhill R. M. Bridson D. M. Verdcourt B. (Rotterdam/Brookfield: Balkema), 703–723.

[B8] Bullock A. A. (1930). Nandi coffee. Bul. Misc. Inform. (Royal Botanic Gardens Kew). 1930 (9), 401–402. doi: 10.2307/4107660

[B9] Busby J. R. (1991). “BIOCLIM – a bioclimatic analysis and prediction system,” in Nature conservation: cost effective biological surveys and data analysis. Eds. Margules C. R. Austin M. P. (Canberra, Australia: Commonwealth Scientific and Industrial Research Organisation (CSIRO), 64–68.

[B10] Carvalho F. G. Sera G. H. Andreazi E. Sera T. de Barista Fonseca I. C. B. Carducci C.. (2017). Drought tolerance in seedlings of coffee genotypes carrying genes of different species. Coffee Sci. 12, 9–16.

[B11] Chadburn H. Davis A. P. (2017a). Coffea canephora . In: The IUCN red list of threatened species 2017. Available at: 10.2305/IUCN.UK.2017-3.RLTS.T18290186A18539466.en (Accessed 19 September 2022).

[B12] Chadburn H. Davis A. P. (2017b). Coffea liberica . In: The IUCN red list of threatened species 2017. Available at: 10.2305/IUCN.UK.2017-3.RLTS.T18537594A18539526.en (Accessed 19 September 2022).

[B13] Charr J.-C. Garavito A. Guyeux C. Crouzillat D. Descombes P. Fournier C.. (2020). Complex evolutionary history of coffees revealed by full plastid genomes and 28,800 nuclear SNP analyses, with particular emphasis on *Coffea canephora* (robusta coffee). Mol. Phyl. Evol. 151, 106906. doi: 10.1016/j.ympev.2020.106906 32653553

[B14] Cheney R. H. (1925). A monograph of the economic species of the genus Coffea L. (The New York University Press).

[B15] Chevalier A. (1903). Notes préliminaires sur qualques caféiers sauvages noveaux ou peu connus de L'Afrique centrale. Rev. Cult. Colon. 12, 256–259.

[B16] Chevalier A. (1929). Les Caféiers du globe, fasc. 1: Généralités sur les caféiers. Ency. Biol. 5, 1–196.

[B17] Chinnappa C. C. (1970). Interspecific hybrids of *Coffea canephora* and *C. liberica* . Genetica. 41, 141–146. doi: 10.1007/BF00958900

[B18] Clarindo W. R. Carvalho C. R. Eveline T. C. Koehler A. D. (2013). Following the track of ‘‘Híbrido de Timor’’ origin by cytogenetic and flow cytometry approaches. Genet. Resour. Crop Evol. 60, 2253–2259. doi: 10.1007/s10722-013-9990-3

[B19] Couturon E. Lashermes P. Charrier A. (1998). First intergeneric hybrids (*Psilanthus ebracteolatus* Hiern × *Coffea arabica* L.) in coffee trees. Can. J. Bot. 76, 542–546. doi: 10.1139/b98-017

[B20] Cramer P. J. S. (1913). Gevens over de variabiliteit van de in nederlandsch-indië verbouwde koffie-sorten. Batavia. Uitgaande Dept. Landbouw Mededeeling. 1, 1–696.

[B21] Cramer P. J. S. (1957). A review of literature of coffee research in Indonesia. (Turrialba: SIC Editorial, Inter-American Institute of Agricultural Sciences).

[B22] Cubry P. De Bellis F. Pot D. Musoli P. Leroy T. (2013). Global analysis of *Coffea canephora* Pierre ex Froehner (Rubiaceae) from the Guineo-Congolese region reveals impacts from climatic refuges and migration effects. Genet. Res. Crop Evol. 60 (2), 483–501. doi: 10.1007/s10722-012-9851-5

[B23] DaMatta F. M. Ramalho J. D. C. (2006). Impacts of drought and temperature stress on coffee physiology and production: a review. Braz. J. Plant Physiol. 18, 55–81. doi: 10.1590/S1677-04202006000100006

[B24] Davis A. P. Bridson D. M. Rakotonasolo F. (2005). “A reexamination of *Coffea* subgenus *Baracoffea* and comments on the morphology and classification of *Coffea* and *Psilanthus* (Rubiaceae-Coffeeae),” in A festschrift for William G. D’Arcy - The legacy of a taxonomist. Monographs in Systematic Botany., 104 (St. Louis: MBG Press).

[B25] Davis A. P. Chadburn H. Moat J. O’Sullivan R. Hargreaves S. Nic Lughadha E. (2019). High extinction risk for wild coffee species and implications for coffee sector sustainability. Sci. Adv. 5, eaav3473. doi: 10.1126/sciadv.aav3473 30746478 PMC6357749

[B26] Davis A. P. Gargiulo R. Almeida I. N. d. M. Caravela M. I. Denison C.. (2021a). Hot coffee: the identity, climate profiles, agronomy, and beverage characteristics of *Coffea racemosa* and *C. zanguebariae* . Front. Sust. Food Syst. 5, 740137. doi: 10.3389/fsufs.2021.740137

[B27] Davis A. P. Gargiulo R. Fay M. F. Sarmu D. Haggar J. (2020). Lost and found: *Coffea stenophylla* and *C. affinis*, the forgotten coffee crop species of West Africa. Front. Plant Sci. 11, 616. doi: 10.3389/fpls.2020.00616 32508866 PMC7248357

[B28] Davis A. P. Govaerts R. Bridson D. M. Stoffelen P. (2006). An annotated taxonomic conspectus of the genus *Coffea* (Rubiaceae). Bot. J. Linn. Soc. 152, 465–512. doi: 10.1111/j.1095-8339.2006.00584.x

[B29] Davis A. P. Kiwuka C. Faruk A. Walubiri M. J. Kalema J. (2022). The re-emergence of Liberica coffee as a major crop plant. Nat. Plants. 8, 1322–1328. doi: 10.1038/s41477-022-01309-5 36522448

[B30] Davis A. P. Mieulet D. Moat J. Sarmu D. Haggar J. (2021b). Arabica-like flavour in a heat-tolerant wild coffee species. Nat. Plants. 7, 413–418. doi: 10.1038/s41477-021-00891-4 33875832

[B31] Davis A. P. Rakotonasolo F. (2021). Six new species of coffee (*Coffea*) from northern Madagascar. Kew Bull. 76, 497–511. doi: 10.1007/s12225-021-09952-5

[B32] Davis A. P. Tosh J. Ruch N. Fay M. F. (2011). Growing coffee: *Psilanthus* (Rubiaceae) subsumed on the basis of molecular and morphological data; implications for the size, morphology, distribution and evolutionary history of *Coffea* . Bot. J. Linn. Soc. 167, 357–377. doi: 10.1111/j.1095-8339.2011.01177.x

[B33] Durand T. De Wildeman E. (1899). Matériaux pour le flore du Congo. Bull. Soc R. Belgique. 38, 171–200.

[B34] Eggeling W. J. Dale I. R. (1952). The indigenous trees of the Ugandan protectorate (Glasgow: Robert MacLehose & Co. Ltd. The University Press).

[B35] Fazouli L. C. Perez Maluf P. Guerreiro Filho O. Medina Filho H. P. Silvarolla M. B. (2000). “Breeding and biotechnology of coffee,” in Coffee biotechnology and quality. Eds. Sera T. Soccol C. R. Pandey A. Roussos S. (Dordrecht: Springer), 27–45.

[B36] Freeman W. Chandler S. (1907). The world's commercial products. (London: Sir Isaac Pitman and Sons, Ltd).

[B37] Gaitán A. L. Cristancho M. A. Castro Caicedo B. L. Rivillas C. A. Cadena Gómez G. (2015). Compendium of coffee diseases and pests. (St. Paul: APS Press).

[B38] Ganesh D. Ram A. S. Prakash N. S. Mishra M. K. Jamsheed A. Jagadeesan M.. (2002). “Evaluation of *Coffea liberica* × *Coffea eugenioides* and its progenies for yield, leaf rust tolerance and quality,” in Proceedings of the 15th Plantation Crops Symposium Placrosym XV, Mysore, India, 10–13 December, 2002. 72–77.

[B39] Garavito A. Montagnon C. Guyot R. Bertrand B. (2016). Identification by the DArTseq method of the genetic origin of the *Coffea canephora* cultivated in Vietnam and Mexico. BMC Plant Biol. 16, 242. doi: 10.1186/s12870-016-0933-y 27814672 PMC5096298

[B40] Gomez C. Dussert S. Hamon P. Hamon S. De Kochko A. Poncet V. (2009). Current genetic differentiation of *Coffea canephora* Pierre ex A.Froehn. in the Guineo-Congolian African zone: cumulative impact of ancient climatic changes and recent human activities. BMC Evol. Biol. 9, 167. doi: 10.1186/1471-2148-9-167 19607674 PMC2717059

[B41] Govaerts R. Rhusam M. Frodin D. Bridson D. Dawson S. Davis A. P. (2022). “ *Coffea* in world checklist of Rubiaceae,” The Royal Botanic Gardens, Kew. Available at: http://wcsp.science.kew.org/. Retrieved 18 July 2022.

[B42] Grisi F. A. Donizeti J. de Castro E. M. de Oliveira C. Biagiotti G. de Melo L. A. (2008). Leaf anatomical evaluations in 'catuaí' and 'siriema' coffee seedlings submitted to water stress. Ciênc. Agrotec. 32, 1730–1736. doi: 10.1590/S1413-70542008000600008

[B43] Haarer A. E. (1962). Modern coffee production. (London: Leonard Hill [Books] Limited).

[B44] Holmgren P. K. Holmgren N. H. Barnett L. C. (1990). “Index herbariorum. part 1,” in The herbaria of the world., 8th edn (New York Botanical Garden: Regnum Vegetabile).

[B45] International Coffee Organization (ICO) (2022). Trade statistics. Available at: http://www.ico.org/trade_statistics.asp (Accessed 7 Aug 2022).

[B46] IUCN Standards and Petitions Subcommittee (2022). Guidelines for using the IUCN red list categories and criteria. (Prepared by the Standards and Petitions Committee of the IUCN Species Survival Comission). Available at: https://www.iucnredlist.org/resources/redlistguidelines. Version 15.1 (July 2022).

[B47] Kalema J. Beentje H. (2012). Conservation checklist of the trees of Uganda. (Kew: Royal Botanic Gardens).

[B48] Kalema J. Hamilton A. (2020). Field guide to the forest trees of Uganda: for identification and conservation. (Croydon: CABI).

[B49] Karger D. N. Conrad O. Böhner J. Kawohl T. Kreft H. Soria-Auza R. W.. (2017). Climatologies at high resolution for the earth’s land surface areas. Sci. Data. 4, 170122. doi: 10.1038/sdata.2017.122 28872642 PMC5584396

[B50] Kassambara A. (2020). Ggpubr: 'ggplot2' based publication ready plots. R package version 0.4.0. Available at: https://CRAN.R-project.org/package=ggpubr.

[B51] Katende A. B. Ssegawa P. Birnie A. Holding C. Tengnäs B. (1999). Wild food plants and mushrooms of Uganda. (Nairobi: Regional Land Management Unit/Sida).

[B52] Kath J. Byrareddy V. M. Craparo A. Nguyen-Huy T. Mushtaq S. Cao L.. (2020). Not so robust: robusta coffee production is highly sensitive to temperature. Glob. Change Biol. 26, 3677–3688. doi: 10.1111/gcb.15097 32223007

[B53] Kiwuka C. Goudsmit E. Tournebize R. de Aquino S. O. Douma J. C. Bellanger L.. (2021). Genetic diversity of native and cultivated Ugandan robusta coffee (*Coffea canephora* Pierre ex A. Froehner): climate influences, breeding potential and diversity conservation. PloS One. 16, e0245965. doi: 10.1371/journal.pone.0245965 33556074 PMC7870046

[B54] Lebrun J. (1941). Recherches morphologiques et systématiques sur les caféiers du Congo. Congo Belge: Publ. L’instit. Nation. L’etude Agron. 11, 1–186.

[B55] Maurin O. Davis A. P. Chester M. Mvungi E. F. Jaufeerally-Fakim Y. Fay M. F. (2007). Towards a phylogeny for *Coffea* (Rubiaceae): identifying well-supported lineages based on nuclear and plastid DNA sequences. Ann. Bot. 100, 1565–1583. doi: 10.1093/aob/mcm257 17956855 PMC2759236

[B56] McCook S. (2014). “Ephemeral plantations: The rise and fall of Liberian coffee 1870–1900,” in Comparing apples, oranges, and cotton. Environmental histories of the plantation. Ed. Uekötter F. (Frankfurt/New York: Campus Verlag), 85–112.

[B57] McCook S. (2019). Coffee is not forever. A global history of coffee leaf rust (Ohio: Ohio University Press).

[B58] Medina Filho H. P. Carvalho A. Medina D. M. (1977a). Germoplasma de *C. racemosa* e seu potencial no melhoramento do cafeeiro. Bragantia. 36, 43–46. doi: 10.1590/S0006-87051977000100040

[B59] Medina Filho H. P. Carvalho A. Monaco L. (1977b). Melhoramento do cafeeiro. XXXVII – observações sobre a resistência do cafeeiro ao bicho mineiro. Bragantia. 36, 131–137. doi: 10.1590/S0006-87051977000100011

[B60] Melo E. F. Fernandes-Brum C. N. Pereira F. J. de Castro E. M. Chalfun-Júnior A. (2014). Anatomic and physiological modifications in seedlings of *Coffea arabica* cultivar Siriema under drought conditions. Ciênc. Agrotec. 38, 25–33. doi: 10.1590/S1413-70542014000100003

[B61] Merot-L'anthoene V. Tournebize R. Darracq O. Rattina V. Lepelley M. Bellanger L.. (2019). Development and evaluation of a genome-wide coffee 8.5K SNP array and its application for high-density genetic mapping and for investigating the origin of *Coffea arabica* L. Pl. Biotechn. J. 17, 1418–1430. doi: 10.1111/pbi.13066 PMC6576098 30582651

[B62] Moat J. Gole T. W. Davis A. P. (2019). Least concern to endangered: applying climate change projections profoundly influences the extinction risk assessment for wild Arabica coffee. Glob. Change Biol. 25, 390–403. doi: 10.1111/gcb.14341 PMC6900256 30650240

[B63] Moat J. Williams J. Baena S. Wilkinson T. Gole T. W. Challa Z. K.. (2017). Resilience potential of the Ethiopian coffee sector under climate change. Nat. Plants. 3, 17081. doi: 10.1038/nplants.2017.81 28628132

[B64] Mulindwa J. Kaaya A. N. Muganga L. Paga M. Musoli P. Sseremba G.. (2022). Cup quality profiles of robusta coffee wilt disease resistant varieties grown in three agro-ecologies in Uganda. J. Sci. Food Agric. 102, 1225–1232. doi: 10.1002/jsfa.11460 34358355

[B65] Musoli P. C. Cilas C. Pot D. Nabaggala A. Nakendo S. Pande J.. (2013). Inheritance of resistance to coffee wilt disease (*Fusarium xylarioides* Steyaert) in robusta coffee (*Coffea canephora* Pierre) and breeding perspectives. Tree Genet. Genomes. 9, 351–360. doi: 10.1007/s11295-012-0557-9

[B66] Musoli C. P. Kangire A. Leroy T. Nabaggala A. Nakendo S. Olal S.. (2009). Towards a variety resistant to coffee wilt disease (CWD) : a case for robusta coffee (Coffea canephora) in Uganda 22nd InternationalConference on coffee science; 14–19 september 2008; campinas, SP,Brazil. (Paris: Association Scientifique Internationale du Café (ASIC), 1472–1479.

[B67] Myers R. Kawabata A. Cho A. Nakamoto S. T. (2020). Grafted coffee increases yield and survivability. Hortic. Tech. 30, 428–432. doi: 10.21273/HORTTECH04550-20

[B68] N'Diaye A. N. Poncet V. Louran J. Hamon S. Noirot M. (2005). Genetic differentiation between *Coffea liberica* var. *liberica* and *C. liberica* var. *dewevrei* and comparison with *C. canephora* . Plant Syst. Evol. 253, 95–104. doi: 10.1007/s00606-005-0300-1

[B69] Nagai C. Rakotomalala J.-J. Katahira R. Li Y. Yamagata K. Ashihara H. (2008). Production of a new low-caffeine hybrid coffee and the biochemical mechanism of low caffeine accumulation. Euphytica. 164, 133–142. doi: 10.1007/s10681-008-9674-9

[B70] Narasimhaswamy R. L. (1960). Arabica selection S.795-its origin and performance – a study. Indian Coffee. 24, 197–204.

[B71] NextGIS (2019). QuickMapServices version 19.11.1. Available at: https://plugins.qgis.org/plugins/quick_map_services/ (Accessed 12 September 2022).

[B72] Ngugi K. Aluka P. (2019). Genetic and phenotypic diversity of robusta coffee (*Coffea canephora* L.). Caffein. Cocoa Based Bever. 8, 89–130. doi: 10.1016/B978-0-12-815864-7.00003-9

[B73] O'Sullivan R. J. Chadburn H. Davis A. P. (2017). Coffea neoleroyi (errata version published in 2020). the IUCN red list of threatened species 2017. Available at: 10.2305/IUCN.UK.2017-3.RLTS.T18290348A176942734.en (Accessed 19 September 2022).

[B74] O'Sullivan R. J. Duarte A. Davis A. P. (2020). Coffea eugenioides (amended version of 2017 assessment). the IUCN red list of threatened species 2020. Available at: 10.2305/IUCN.UK.2020-2.RLTS.T18290318A174150624.en (Accessed 03 August 2022).

[B75] Panaligan A. C. Baltazar M. D. Alejandro G. J. D. (2020). Genetic polymorphism of registered and popularly cultivated coffee (*Coffea* spp.) in the Philippines using inter-simple sequence repeats markers. Biodiversitas. 21, 4228–4233. doi: 10.13057/biodiv/d210938

[B76] Panaligan A. C. Baltazar M. D. Alejandro G. J. D. (2021). Molecular authentication of commercially cultivated coffee (*Coffea* spp.) in the Philippines using DNA barcodes. Int. J. Agric. Biol. 25, 27−230. doi: 10.17957/IJAB/15.1660

[B77] Phiri N. Baker P. S. (2009). Coffee wilt in Africa. Final technical report of the regional coffee wilt programme 2000–07). (CAB International).

[B78] QGIS Development Team (2022). QGIS geographic information system. (Open Source Geospatial Foundation Project). Available at: http://qgis.osgeo.org.

[B79] R Core Team (2020). R: A language and environment for statistical computing. (Vienna, Austria: R Foundation for Statistical Computing). Available at: https://www.R-project.org/.

[B80] Reddy A. G. S. Raju K. V. V. S. N. Dharmaraj P. S. (1985). Breeding behaviour of ligenioides — a spontaneous amphidiploid between *Coffea liberica* and *C. eugenioides* . J. Coffee Res. 15, 33–37.

[B81] Rutherford M. A. (2006). Current knowledge of coffee wilt disease, a major constraint to coffee production in Africa. Phytopathology. 96, 663–666. doi: 10.1094/PHYTO-96-0663 18943187

[B82] Stoffelen P. (1998). Coffea and Psilanthus in Tropical Africa: A systematic and palynological study, including a revision of the West and central African species. (Katholieke Universiteit Leuven).

[B83] Surya Prakash N. Combes M.-C. Somanna N. Lashermes P. (2002). AFLP analysis of introgression in coffee cultivars (*Coffea arabica* L.) derived from a natural interspecific hybrid. Euphytica. 124, 265–271. doi: 10.1023/A:1015736220358

[B84] Thiers B. (2019). Index herbariorum: A global directory of public herbaria and associated staff. (New York Botanical Garden's Virtual Herbarium). Available at: http://sweetgum.nybg.org/science/ih/ (Accessed 11 September 2019).

[B85] Thomas A. S. (1935). Types of robusta coffee and their selection in Uganda. East Afr. Agr. J. 1, 193–197. doi: 10.1080/03670074.1935.11663646

[B86] Thomas A. S. (1940a). “Section IX. a. robusta coffee,” in Agriculture in Uganda. Ed. Tothill J. D. (London: Oxford University Press), 289–311.

[B87] Thomas A. S. (1940b). “Section IX. b. other coffees,” in Agriculture in Uganda. Ed. Tothill J. D. (London: Oxford University Press), 311–313.

[B88] Thomas A. S. (1940c). “Section x. Arabica coffee. i. history and general,” in Agriculture in Uganda. Ed. Tothill J. D. (London: Oxford University Press), 314–325.

[B89] Thomas A. S. (1942). The wild Arabica coffee on the Boma Plateau. Anglo-Egyptian Sudan. Empire J. Expt. Agric. 10, 207–212.

[B90] Thomas A. S. (1944). The wild coffees of Uganda. Empire J. Expt. Agric. 12, 1–12.

[B91] Tothill J. D. (1940). Agriculture in Uganda. (London: Oxford University Press).

[B92] Uganda Coffee Development Authority (UCDA) (2017). Uganda — country coffee profile. Available at: https://ugandacoffee.go.ug/resource-center/publications.

[B93] Venancio L. P. Filgueiras R. Mantovani E. C. do Amaral C. H. da Cunha F. F. dos Santos Silva F. C.. (2020). Impact of drought associated with high temperatures on *Coffea canephora* plantations: a case study in espírito Santo state, Brazil. Sci. Rep. 10, 19719. doi: 10.1038/s41598-020-76713-y 33184345 PMC7665182

[B94] Wellman F. L. (1961). Coffee: Botany, cultivation and utilization. (London: Leonard Hill [Books] Limited; New York: Interscience Publishers, Inc).

[B95] Wickham H. (2016). ggplot2: Elegant graphics for data analysis. (Springer-Verlag New York).

[B96] Wrigley G. (1988). Coffee – tropical agriculture series. (Harlow: Longman Scientific & Technical).

[B97] Yonas B. Bayetta B. Chemeda F. (2014). Assessments of resistances of indigenous Arabica coffee genotypes for multiple diseases. J. Plant Breed. Crop Sci. 2, 54–61. doi: 10.15580/GJPBCS.2014.3.101013898

